# Inhibition of histone deacetylation rescues phenotype in a mouse model of Birk-Barel intellectual disability syndrome

**DOI:** 10.1038/s41467-019-13918-4

**Published:** 2020-01-24

**Authors:** Alexis Cooper, Tamer Butto, Niklas Hammer, Somanath Jagannath, Desiree Lucia Fend-Guella, Junaid Akhtar, Konstantin Radyushkin, Florian Lesage, Jennifer Winter, Susanne Strand, Jochen Roeper, Ulrich Zechner, Susann Schweiger

**Affiliations:** 1 Institute of Human Genetics, University Medical Center of the Johannes Gutenberg University Mainz, Langenbeckstr. 1, 55131 Mainz, Germany; 2 Institute for Developmental Biology and Neurobiology, Johannes Gutenberg University Mainz, Staudingerweg 9, 55128 Mainz, Germany; 3 Institute of Neurophysiology, Goethe University Frankfurt, Theodor-Stern-Kai 7, 60590 Frankfurt, Germany; 4 Focus Program Translational Neuroscience, Center for Rare Diseases, University Medical Center of the Johannes Gutenberg University Mainz, Langenbeckstr. 1, 55131 Mainz, Germany; 5 Université Côte d’Azur, INSERM, Centre National de la Recherche Scientifique, Institut de Pharmacologie Moléculaire et Cellulaire, Labex ICST, 660, route des Lucioles Sophia Antipolis, 06560 Valbonne, France; 6 Department of Internal Medicine I, University Medical Center of the Johannes Gutenberg University Mainz, Obere Zahlbacher Straße 63, 55131 Mainz, Germany; 7 Senckenberg Center of Human Genetics, Weismüllerstraße 50, 60314 Frankfurt, Germany; 8 Center for Orphan Diseases of the Central Nervous System, University Medical Center of the Johannes Gutenberg University Mainz, Langenbeckstr. 1, 55131 Mainz, Germany; 9 German Resilience Centre, University Medical Center of the Johannes Gutenberg University Mainz, Langenbeckstr. 1, 55131 Mainz, Germany

**Keywords:** Epigenetics, Neurodevelopmental disorders, Developmental disorders

## Abstract

Mutations in the actively expressed, maternal allele of the imprinted *KCNK9* gene cause Birk-Barel intellectual disability syndrome (BBIDS). Using a BBIDS mouse model, we identify here a partial rescue of the BBIDS-like behavioral and neuronal phenotypes mediated via residual expression from the paternal *Kcnk9* (*Kcnk9*^pat^) allele. We further demonstrate that the second-generation HDAC inhibitor CI-994 induces enhanced expression from the paternally silenced *Kcnk9* allele and leads to a full rescue of the behavioral phenotype suggesting CI-994 as a promising molecule for BBIDS therapy. Thus, these findings suggest a potential approach to improve cognitive dysfunction in a mouse model of an imprinting disorder.

## Abstract (web summary)

Birk-Barel intellectual disability is an imprinting syndrome due to maternally-only transmitted mutations of *KCNK9/TASK3*. Here authors are using a heterozygous deletion of the active maternal *Kcnk9* allele to model the disease and show phenotypic rescue by HDAC inhibition.

## Introduction

Birk-Barel intellectual disability (ID) dysmorphism syndrome (BBIDS or *KCNK9* imprinting syndrome, OMIM 612292) is typically associated with congenital central hypotonia, developmental delay, intellectual disability, severe feeding problems, and hyperactivity^1,2^. The disease is inherited autosomal dominantly with maternal-only transmission^1^, as the *KCNK9* gene is embryonically paternally silenced (imprinted) in man and mouse. It encodes the potassium channel subunit TASK3, which dimerizes to form two-pore domain potassium (K2P) leak channels^3^. The mouse *Kcnk9* gene maps to an imprinted cluster on mouse chromosome 15 together with further imprinted genes, i.e., the brain-specific maternally expressed genes *Ago2*, *Chrac1*, and *Trappc9* and the paternally expressed *Peg13* gene^4^.

*Kcnk9* mRNA expression is widespread in the central nervous system^5,6^; in rodents with notably high levels in cerebellar granule neurons, the locus coeruleus (LC), the dorsal raphe nuclei, hippocampal CA1 and CA3 pyramidal neurons, and several hypothalamic nuclei^7,8^. Homozygous deletion of *Kcnk9* in the mouse (*Kcnk9*KO^hom^) reduces the resting potassium conductance and enhances spike firing accommodation in adult cerebellar granule neurons^9^. *Kcnk9*KO^hom^ mice further display increased nocturnal motor activity, cognitive deficits, as well as a reduced sensitivity to inhalation anesthetics and the cannabinoid receptor agonist WIN55212-2 mesylate ^10–12^. More recently, RNAi-based knockdown of *Kcnk9* and expression of a dominant-negative mutant KCNK9, which had been associated with the human disease phenotype were shown to impair neuronal migration during mouse cortical development^13^. However, the phenotype of mice with heterozygous deletion of the active maternal *Kcnk9* allele (*Kcnk*9KO^mat^) thus mimicking BBIDS has not yet been characterized.

DNA methylation is well established as a key player in imprinting regulation by multiple studies, which characterized the kinetics and mechanisms of methylation reprogramming at imprinting control regions (ICRs). The critical involvement of post-translational histone modifications in transcriptional regulation, in general, is also well accepted, but much less explored for ICRs^14^. It is assumed that an interplay between DNA methylation and histone acetylation is essential for proper erasure and resetting of imprints in the germline as well as selective imprint maintenance during postzygotic reprogramming^15^. A differentially methylated region (DMR) which is methylated on the maternal allele and assumed to be involved in *Kcnk9* gene regulation has been only identified in the promoter region of *Peg13*^16,17^. Promoter CpG islands of *Kcnk9* are unmethylated, but display high levels of active histone H3 lysine 4 monomethylation (H3K4me1) and histone H3 lysine 27 acetylation (H3K27ac) chromatin marks in brain tissues^4^.

Here, we characterize the behavioral and neuronal phenotype of mice with heterozygous deletion of the active maternal *Kcnk9* allele (*Kcnk9*KO^mat^). We demonstrate partial behavioral rescue in these animals compared with full knockout animals and show that epigenetic manipulation stimulates *Kcnk9*^pat^ expression sufficiently, to rescue the behavioral phenotypes, thereby opening new avenues for treatment of cognitive dysfunctions in BBIDS.

## Results

### Deletion of *Kcnk9* leads to impaired behavior

**Fig. 1:**
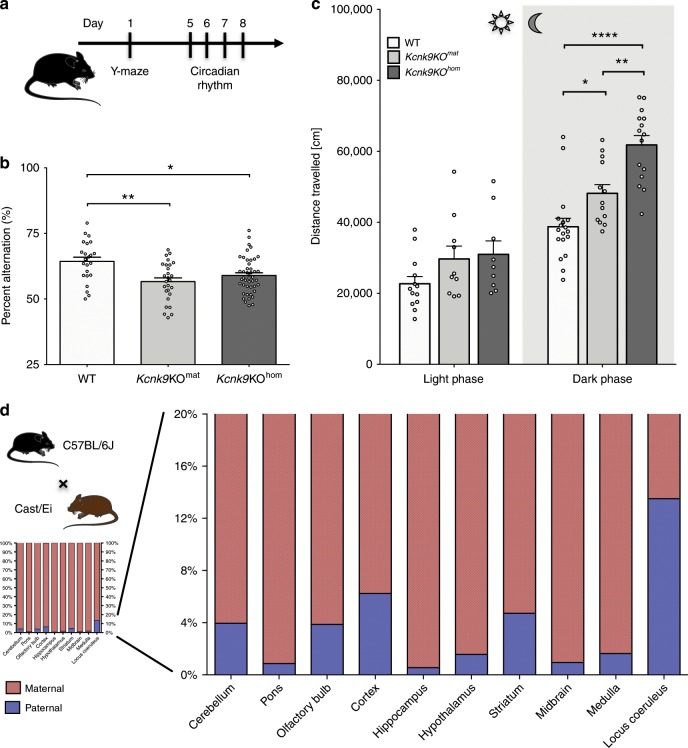
Deletion of non-canonical imprinted *Kcnk9* gene leads to impaired behavior of *Kcnk9*KO mice. **a** Schematic representation of the sequence of mouse behavioral experiments. **b** Y-maze percentage alternation analysis of WT (*n* = 23), *Kcnk9*KO^mat^ (*n* = 27), and *Kcnk9*KO^hom^ (*n* = 44) mice. A spontaneous alternation was defined as consecutive entries into all three arms without revisiting an arm. *Kcnk9*KO mice display a significant decrease in percentage alteration compared with WT mice. One-way ANOVA: *F*(2, 91) = 7.261, *P* = 0.0012; followed by Bonferroni’s multiple comparison post hoc test, *P < 0.05, **P < 0.01. **c** Total locomotor activity (distance traveled in the home cage) in light (12 h, sun symbol)/dark (12 h, moon symbol) phase. The left section shows no difference in distance traveled in the light phase of WT (*n* = 13), *Kcnk9*KO^mat^ (*n* = 10) and *Kcnk9*KO^hom^ (*n* = 9) mice. One-way ANOVA: *F*(2, 29) = 2.281, *P* = 0.1203. The right section depicts the nocturnal activity of WT (*n* = 18), *Kcnk9*KO^mat^ (*n* = 13) and *Kcnk9*KO^hom^ (*n* = 15) mice. *Kcnk9*KO^mat^ and *Kcnk9*KO^hom^ mice display significantly increased nocturnal hyperactivity compared with WT littermates with activity scores of *Kcnk9*KO^mat^ mice intermediate between *Kcnk9*KO^hom^ and WT mice. One-way ANOVA: *F*(2, 43) = 22.70, *P* < 0.0001; followed by Bonferroni’s multiple comparison post hoc test, *P < 0.05, **P < 0.01, ****P *<* 0.0001. **b**, **c** Behavioral experiments were performed in seven independent sessions. **d** Non-canonical *Kcnk9* imprinting in (C57BL/6xCast/Ei)F1 hybrid mice. Quantification of Allele-Specific Expression by Pyrosequencing (QUASEP) of several brain regions from (C57BL/6xCast/Ei)F1 hybrid mice; maternal allele (red) and paternal allele (blue). Cerebellum *n* = 14, pons *n* = 10, olfactory bulb *n* = 12, cortex *n* = 14, hippocampus *n* = 14, hypothalamus *n* = 9, striatum *n* = 5, midbrain *n* = 5, medulla *n* = 5, and locus coeruleus *n* = 4; *n* = biologically independent samples. **a**–**d** Data are means ± 1 SEM (standard error of the mean). Statistical analyses and approaches are provided in Supplementary Table 1. Source data are provided as a Source Data file. The mouse images in this figure were created using Servier Medical Art templates, which are licensed under a Creative Commons Attribution 3.0 Unported License; https://smart.servier.com.

To assess behavioral deficits along the BBIDS phenotype in *Kcnk9*KO mice, we performed in vivo experiments in adult wild type (WT) as well as *Kcnk9*KO^mat^ and *Kcnk9*KO^hom^ mice with a deletion of *Kcnk9* exon 2 as previously described^18^ (Fig. 1a). Following the strictly monoallelic expression pattern of *Kcnk9* in mouse brain (<1% paternal expression)^3^, we expected largely concordant phenotypes in mice carrying a deletion of both *Kcnk9* alleles (*Kcnk9*KO^hom^) or only the maternal *Kcnk9* allele (*Kcnk9*KO^mat^), respectively.

No differences in behavior between *Kcnk9*KO^hom^, *Kcnk9*KO^mat^, and WT littermates were seen in elevated plus maze and open field testing for anxiety (Supplementary Fig. 1a, b) and in the rotarod-test for motor coordination (Supplementary Fig. 1c). Given the described intellectual disability in BBIDS patients, we then examined whether working memory was affected in *Kcnk9*KO mice. Spontaneous alternation relies on the natural tendency of rodents to explore a novel environment and can be quantified using a Y-maze task. The ability to remember the immediately preceding choice is considered an indicator for active working memory. In the Y-maze, spontaneous alternation was defined as consecutive entries into all three arms without revisiting an arm. The average percentage of spontaneous alternation made by *Kcnk9*KO^hom^, *Kcnk9*KO^mat^, and WT mice across a 10-min trial was analyzed and revealed a significant reduction of spontaneous alternation by about 10% in both *Kcnk9*KO^hom^ and *Kcnk9*KO^mat^ mice compared with WT littermates (Fig. 1b). There was no significant difference in the extent of the working memory impairment between *Kcnk9*KO^hom^ and *Kcnk9*KO^mat^ mice (Fig. 1b). Our findings indicate an impaired working memory in *Kcnk9*KO^hom^ and *Kcnk9*KO^mat^ mice, which is consistent with previously reported observations in *Kcnk9*KO^hom^ mice^10^.

Circadian rhythms in mammals are endogenously coordinated oscillations of biological parameters such as the sleep/wake cycles with an overall period length of about 24 h^19^. KCNK9 channels are expected to contribute to the in vivo electrical activity of neurons in those brain regions associated with the regulation of circadian rhythms and arousal^10–12,20^. Spontaneous motor activity analysis during light (resting) and dark (active) phase was performed, resembling day and night in diurnal humans, respectively. A significantly increased overall locomotor activity during the dark phase was found in *Kcnk9*KO^hom^ and *Kcnk9*KO^mat^ mice compared with WT controls, describing an exaggerated nocturnal activity in *Kcnk9*KO animals (Fig. 1c), which is in consensus with previous observations^10^. Surprisingly, an intermediate phenotype for dark phase activity was observed in the *Kcnk9*KO^mat^ animals with statistically significant differences to both WT and *Kcnk9*KO^hom^ animals. No significant differences between genotypes were observed in the overall locomotor activity during the light phase (Fig. 1c, Supplementary Table 1).

Together, these data demonstrate that the loss of *Kcnk9* in mice results in the impairment of behavioral parameters resembling components of the BBIDS phenotype. Interestingly, we found an intermediate phenotype for nocturnal locomotor activity in animals with a loss of only the actively expressed maternal allele (*Kcnk9*KO^mat^) suggesting the involvement of the silenced paternal allele.

### Non-canonical imprinting of *Kcnk9* in the mouse brain

In human and mouse brain *KCNK9* was reported to be monoallelically expressed from the maternal allele^3,4^ while the paternal allele is silenced. To elucidate if the intermediate phenotype of *Kcnk9*KO^mat^ animals was due to residual expression from the paternal allele, we analyzed the parent-of-origin allele-specific expression pattern of *Kcnk9* in different brain regions of F1 hybrid animals from crosses between C57BL/6 (B6) and Mus musculus castaneus (Cast/Ei) mouse strains [(C57BL/6xCast/Ei)F1]^3^. As expected, we observed a predominant expression of the maternal *Kcnk9* allele in all analyzed brain regions (Fig. 1d). However, we also detected significant expression from the repressed paternal allele, which represented 1–14% of all transcripts depending on the brain region analyzed (Fig. 1d, suppl. Fig. 2c). Highest paternal expression was observed in the LC (Fig. 1d). Accuracy of the LC tissue-punches was demonstrated through elevated gene expression levels of tyrosine hydrolase (TH) in LC samples (Supplementary Fig. 2a, b).

These data demonstrate a brain region-specific leakiness of the imprint on the *Kcnk9*^pat^ allele with a peak in the LC. This leakiness might be responsible for the observed intermediate phenotype in *Kcnk9*KO^mat^ animals and suggested a particular importance of LC *Kcnk9* expression for locomotor activity during the active (dark) phase.

### *Kcnk9* knockdown in the LC induces elevated nocturnal activity

**Fig. 2:**
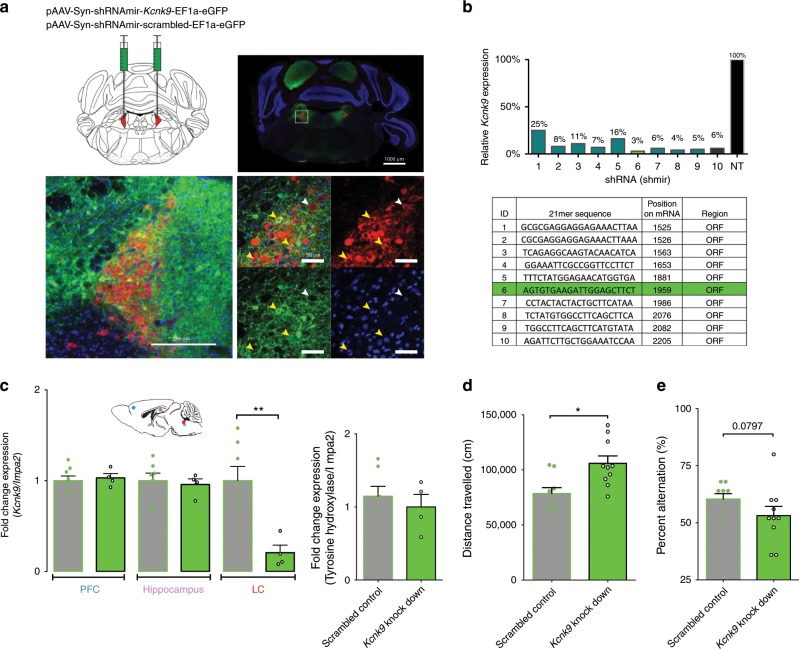
*Kcnk9* knockdown in the locus coeruleus induces elevated nocturnal activity. **a** Bilateral virus injection with pAAV-Syn-shRNAmir-Kcnk9-EF1a-eGFP (KD) or Syn-shRNAmir-scrambled-EF1a-eGFP (SC) in the locus coeruleus (LC): Top left: schematic coronal section of the mouse brain at the position −5.4 relative to bregma^43^; red triangles represent the LC; top right: Immunofluorescence staining of a coronal section. Scale represents 1000 µm (4×). Bottom left: Images of the LC. Scale represents 200 µm (20×); bottom right: Magnification of LC cells. Scale represents 50 µm (60×), majority of tyrosine hydroxylase (TH; red) expressing cells co express GFP (green) (yellow arrows), while also GFP negative TH expressing cells could be observed (white arrow). Blue, DAPI; green, GFP; red, TH (TH serves as a norepinephrine marker). **b** Cell-based quantification of *Kcnk9* gene knockdown (*Kcnk9* KD) using *Kcnk9*-specific shRNAs by RT-qPCR compared with negative control (NT). Validated shRNAs with KD > 80%: 2, 3, 4, 5, 6, 7, 8, 9, and 10. ShRNA 6 was utilized for AAV generation in 293T cells. Arithmetic means of *Kcnk9* expression of presented IDs were provided by Sirion Biotech. **c** Left: *Kcnk9* knockdown efficiency validation in vivo. *Kcnk9* expression analysis in the prefrontal cortex (PFC, blue circle, SC *n* = 8, KD *n* = 4), hippocampus (pink circle, SC *n* = 7, KD *n* = 4) and LC (red circle, SC *n* = 7, KD *n* = 4) by RT-qPCR revealed a significant down-regulation of *Kcnk9* gene expression in the LC of *Kcnk9* KD mice compared with mice injected with scrambled controls. Mann–Whitney U: *P* = 0.004. Right: Tyrosine hydroxylase (TH) RT-qPCR expression analysis in the LC to validate accuracy of tissue collection. LC samples of *Kcnk9* KD (*n* = 4) and scrambled controls (*n* = 7) exhibit similar TH levels demonstrating sample collection accuracy. *n* = biologically independent mice. **d** Total locomotor activity in dark (12 h) phase, 21 days after AAV-injections. Mice injected with pAAV-Syn-shRNAmir-*Kcnk9*-EF1a-eGFP (*n* = 10) display increased nocturnal activity compared with pAAV-Syn-shRNAmir-scambled-EF1a-eGFP (*n* = 9) injected controls. Mann–Whitney U: *P* = 0.0101. **e** Y-maze percentage alternation analysis reveals tendency toward impaired working memory in *Kcnk9* KD mice (*n* *=* 10) compared with age-matched controls (*n* = 10); *n* = biologically independent mice. Mann–Whitney U: *P* = 0.0797. Values are means ± SEM. Statistical analyses and approaches are provided in Supplementary Table 1. Source data are provided as a Source Data file.

To test the role of functional *Kcnk9* expression in LC neurons for the control of nocturnal activity, we bilaterally infused AAV vectors for expression of eGFP and shRNAmir sequences, either scrambled (pAAV-Syn-shRNAmir-scrambled-EF1a-eGFP) or specifically targeting *Kcnk9* mRNA (pAAV-Syn-shRNAmir-*Kcnk9*-EF1a-eGFP), into the LC of WT mice under stereotactic control (Fig. 2a). Efficiency of the used shRNA sequence was pre-tested in vitro (Fig. 2b) and validated by RT-qPCR from mouse brain tissue (Fig. 2c). Targeting efficiency and selectivity of stereotactically guided infusion was demonstrated by eGFP immunohistochemistry (Fig. 2a). Twenty-one days after injection, animals were tested for circadian activity and working memory. A significant and selective increase of nocturnal activity was detected in the shRNAmir-*Kcnk9* injected animals compared with age-matched control animals injected with the scrambled shRNAmir virus (Fig. 2d). These results demonstrated that altering local expression of *Kcnk9* in the LC was sufficient to selectively affect dark-phase activity in mice. Furthermore, it identified LC as an important neural hub for mediating the behavioral effects of altered *Kcnk9* expression, which warranted further mechanistic analysis of these neurons. Interestingly, a clear trend toward impaired working memory was also observed in shRNAmir-*Kcnk9* injected animals (Fig. 2e), suggesting that *Kcnk9* expression also controls working memory-related activity of LC neurons.

### Increased dark-phase LC pacemaking activity in *Kcnk9*KO^hom^ but not *Kcnk9*KO^mat^ mice

Electrical activity of LC neurons drives periods of wakefulness and arousal^21–23^. Indeed, selective optogenetic activation of LC neurons revealed their causal role in sleep-to-wake transitions and locomotor arousal by demonstrating that sustained 3-Hz LC neuronal stimulation enhanced spontaneous locomotion (total track length) by about 50%^24^. As we detected a similar increase of spontaneous locomotion during the active (dark) phase of *Kcnk9*KO^hom^ mice compared with WT controls, we reasoned that KCNK9-dependent differences in LC pacemaking activity might contribute to this phenotype.

**Fig. 3:**
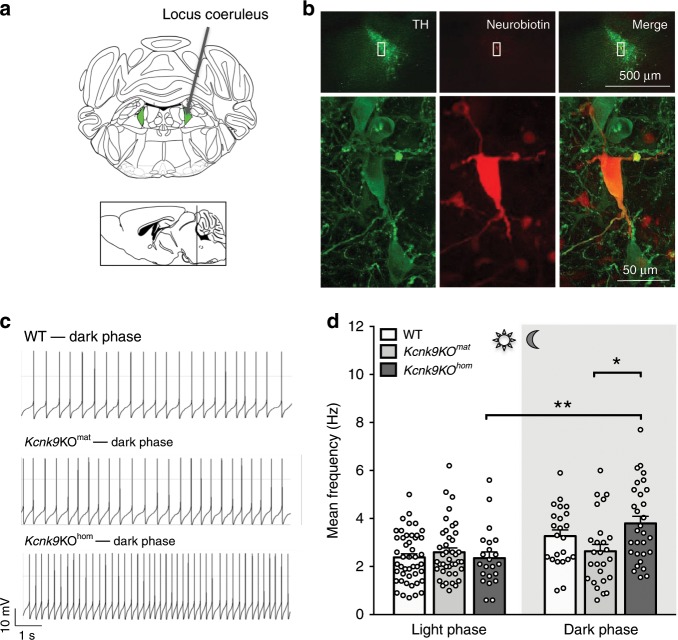
Electrophysiological analysis of LC neurons. **a** Schematic overview of the locus coeruleus (LC) and its position in the adult mouse brain, bregma −5.40 and −5.52 (the coordinates were according to the mouse brain atlas^43)^. Green triangles represent the LC. **b** Confocal images showing TH-positive signal (green), neurobiotin-positive signal (red) and a merge of both signals (yellow). Upper images show an overview of the LC at a low-magnification (×4) and lower images display signals of analyzed single-cell at high-magnification (×60 oil). **c** Electrophysiological traces of single cells in the night phase of WT, *Kcnk9*KO^mat^ and *Kcnk9*KO^hom^ animals. Recordings from synaptically isolated spontaneously active LC neurons reveal increased pacemaker frequency in LC neurons from *Kcnk9*KO^hom^ mice during dark phase. **d** Scatter dot-plot of mean frequencies including all analyzed cells in the day and night phase of WT (light *n* = 46, *N* = 5, dark *n* = 23, *N* = 3), *Kcnk9*KO^mat^ (light *n* = 41, *N* = 5, dark *n* = 26, *N* = 4) and *Kcnk9*KO^hom^ (light *n* = 22, *N* = 3, dark *n* = 30, *N* = 5) animals. Ordinary one-way ANOVA with Bonferroni’s multiple comparison post hoc test, *P < 0.05, **P < 0.01. Values are means ± SEM. *N* = number of mice, *n* = total number of cells. Statistical analyses and approaches are provided in Supplementary Table 1. Source data are provided as a Source Data file.

By recording from synaptically isolated (including inhibition of somatodendritic alpha2-autoreceptors), spontaneously active LC neurons in brainstem slices from adult mice, we observed a dark phase-selective, about 70% increase of pacemaker frequency in LC neurons from *Kcnk9*KO^hom^ mice (Fig. 3a–d). In contrast, no significant dark-phase increase of LC pacemaker frequency was observed in WT animals (Fig. 3c, d). These results demonstrate that some degree of KCNK9 channel expression is necessary to selectively dampen enhanced pacemaker activity during the dark phase, which is present in *Kcnk9*KO^hom^ mice. Interestingly, LC neurons from *Kcnk9*KO^mat^ animals displayed no significant increase in pacemaker activity in the dark phase (Fig. 3c, d), but a significant difference in pacemaker activity in the dark was observed between *Kcnk9*KO^hom^ and *Kcnk9*KO^mat^ animals. This indicated that a certain level of functional expression of KCNK9 channels from the *Kcnk9*^pat^ allele in LC is present in *Kcnk9*KO^mat^ animals to prevent the full nocturnal in vitro pacemaker frequency increase. This level of *Kcnk9* expression might at the same time not be sufficient to dampen in vivo LC activity, which is also driven by synaptic inputs from neuronal networks, in line with the observed intermediate behavioral phenotype regarding dark-phase locomotion in *Kcnk9*KO^mat^ mice. In line with this argument, we show below that boosting further expression of KCNK9 channel subunits from paternal alleles indeed also restores the WT motor activity phenotype.

### CI-994 activates the paternally repressed *Kcnk9* allele

The paternally inherited *Kcnk9/KCNK9* gene is epigenetically silenced yet structurally unimpaired. Our behavioral and gene expression experiments suggest a contribution of paternally expressed *Kcnk9* to the intermediate phenotype of the *Kcnk9*KO^mat^ mice and the possibility of upregulation of the paternal allele in the case of loss of the maternal allele. We, therefore, speculated that exogenous application of epigenetic modulators might alter the structure at the *Kcnk9* promoter region and result in further derepression of the *Kcnk9*^pat^ allele. We hypothesized that this could fully compensate for the loss of the maternal allele and rescue the BBIDS-like phenotype of *Kcnk9*KO^mat^ mice.

**Fig. 4:**
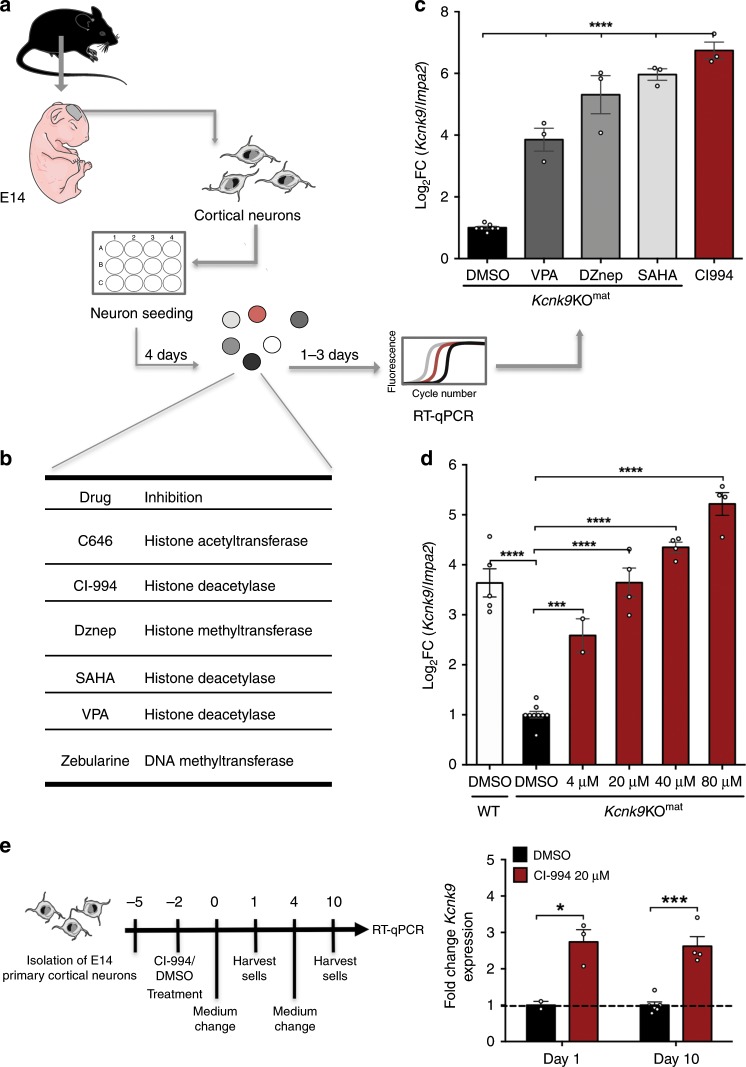
Identification of epigenetic modulators upregulating *Kcnk9*^pat^ expression in mPCNs. **a** Workflow of murine primary cortical neurons (mPCN) isolation, drug treatment, and RT-qPCR analysis. **b** Epigenetic modulators used for treatment of mPCNs (left column) with enzymatic activities inhibited by them (right column). **c** Significant upregulation of paternal allele-derived *Kcnk9* mRNA in DZnep-, SAHA-, VPA- and CI-994-treated E14 *Kcnk9*KO^mat^ mPCNs compared with DMSO-treated *Kcnk9*KO^mat^ mPCNs detected by RT-qPCR (3-day treatment, normalization to *Impa2*, DMSO *n* = 7 cultures/group, DZnep (20 µM) SAHA (30 µM), VPA (5 mM) and CI-994 (40 µM) *n* = 3 cultures/group). One-way ANOVA: F(5, 22) = 75.51, *P* < 0.0001; followed by Bonferroni’s multiple comparison post hoc test to *Kcnk9*KO^mat^ DMSO control, ****P < 0.0001. **d** Upregulation of paternal allele-derived *Kcnk9* mRNA after treatment of *Kcnk9*KO^mat^ mPCNs with different CI-994 concentrations compared with paternal allele-derived *Kcnk9* mRNA of DMSO-treated *Kcnk9*KO^mat^ mPCNs and *Kcnk9* mRNA of DMSO-treated WT mPCNs detected by RT-qPCR (1 day treatment, normalization to *Impa2*, *Kcnk9*KO^mat^ DMSO *n* = 9; 4 µM *n* = 2; 20–80 µM *n* = 4 and WT DMSO *n* = 5 cultures/group). One-way ANOVA: *F*(5, 22) = 75.51, *P* < 0.0001; corrected with Bonferroni’s multiple comparison post hoc test to *Kcnk9*KO^mat^ DMSO mPCNs, ***P < 0.001****, *P* < 0.0001, **c**, **d** performed in 2–4 independent experiments. **e** Duration of *Kcnk9* derepression in vitro. Workflow is illustrated in schematical representation (left). After 1 and 10 days, *Kcnk9* is significantly upregulated after CI-994 treatment in mPCN compared with DMSO controls. One day DMSO *n* = 2, 1 day CI-994 *n* = 3, day 10 DMSO *n* = 6, day 10 CI-994; *n* = 4; *n* = samples (3–5 wells/sample; 6 embryos pooled). Day 1: *P* = 0.0297, day 10: *P* = 0.0001, Mann–Whitney U. **c**–**e** Values are means ± SEM. Statistical analyses and approaches are provided in Supplementary Table 1. Source data are provided as a Source Data file. Images displaying the mouse, the embryo and the neurons in this figure were created using Servier Medical Art templates, which are licensed under a Creative Commons Attribution 3.0 Unported License; https://smart.servier.com.

To investigate the paternally derived *Kcnk9* transcript levels after epigenetic drug treatment, we isolated E14 mouse primary cortical neurons (mPCNs) from WT and *Kcnk9*KO^mat^ animals from crosses between WT males and *Kcnk9*KO^hom^ females (Fig. 4a). We then chose six different compounds representing different classes of epigenetic modulators for treatment (Fig. 4b).

Murine PCNs were treated for 3 days with the epigenetic modulators and *Kcnk9* expression was analyzed by RT-qPCR. mPCNs treated with DZNep (20 µM), SAHA (30 µM), VPA (5 mM), and CI-994 (40 µM) exhibited a significantly increased *Kcnk9*^pat^ expression (RT-qPCR) compared with *Kcnk9*KO^mat^ mPCNs treated with dimethyl sulfoxide (DMSO) (control vehicle) (Fig. 4c). By contrast, treatment with the compounds Zebularine and C646 did not show any differential *Kcnk9* expression in *Kcnk9*KO^mat^ mPCNs compared with control conditions (Supplementary Fig. 3a).

For further investigations, we focused on the benzamide-based second-generation histone deacetylase inhibitor (HDACi) CI-994, a selective inhibitor of class I HDACs, which is a potent inhibitor of HDAC1 and 3 isoenzymes relative to HDAC6 and HDAC8^25^. CI-994 showed the most efficient upregulation of *Kcnk9*^pat^ expression in *Kcnk9*KO^mat^ mPCNs (Fig. 4c). Furthermore, a previous study reported that intraperitoneal administration of CI-994 in WT mice resulted in long-lasting CI-994 levels in the brain without affecting the overall behavioral phenotype of mice^26^.

The effect of CI-994 in *Kcnk9*KO^mat^ mPCNs was further tested in a dose-response experiment. *Kcnk9*KO^mat^ mPCNs were treated with increasing concentrations of CI-994 for 24 h, and *Kcnk9* expression was analyzed using RT-qPCR in comparison to DMSO-treated *Kcnk9*KO^mat^ and WT control cells (Fig. 4d). As expected, the *Kcnk9*^pat^ expression in DMSO-treated *Kcnk9*KO^mat^ mPCNs was significantly reduced compared with that of WT mPCNs. After CI-994 treatment of *Kcnk9*KO^mat^ mPCNs, we observed a strong linear correlation between the increase of *Kcnk9*^pat^ expression and CI-994 dosage suggesting a specific, dose-dependent effect of CI-994. Notably, *Kcnk9* expression in 80 µM CI-994-treated *Kcnk9*KO^mat^ mPCNs exceeded that of DMSO-treated WT mPCNs in vitro (Fig. 4d). Treatment of *Kcnk9*KO^mat^ mPCNs with 20 µM CI-994 showed a consistent increase of *Kcnk9*^pat^ expression even after 10 days suggesting a prolonged and stable in vivo effectiveness (Fig. 4e). Cell viability analysis after treatment of *Kcnk9*KO^mat^ mPCNs with 10 or 20 µM CI-994 did not reveal any toxic effect (Supplementary Fig. 4c). Intriguingly, CI-994 treatment did not affect the expression of other nearby imprinted genes within the imprinted cluster on mouse chromosome 15 in vitro (Supplementary Fig. 3b).

### CI-994 rescues the behavioral phenotype of *Kcnk9*KO^mat^ animals

The detection of *Kcnk9*^pat^ allele expression in several brain regions, prominently among them the LC, as well as the observation of intermediate phenotypes in *Kcnk9*KO^mat^ animals led us to hypothesize that epigenetic manipulation could further stimulate paternal gene expression and thereby boost the phenotypical rescue.

**Fig. 5:**
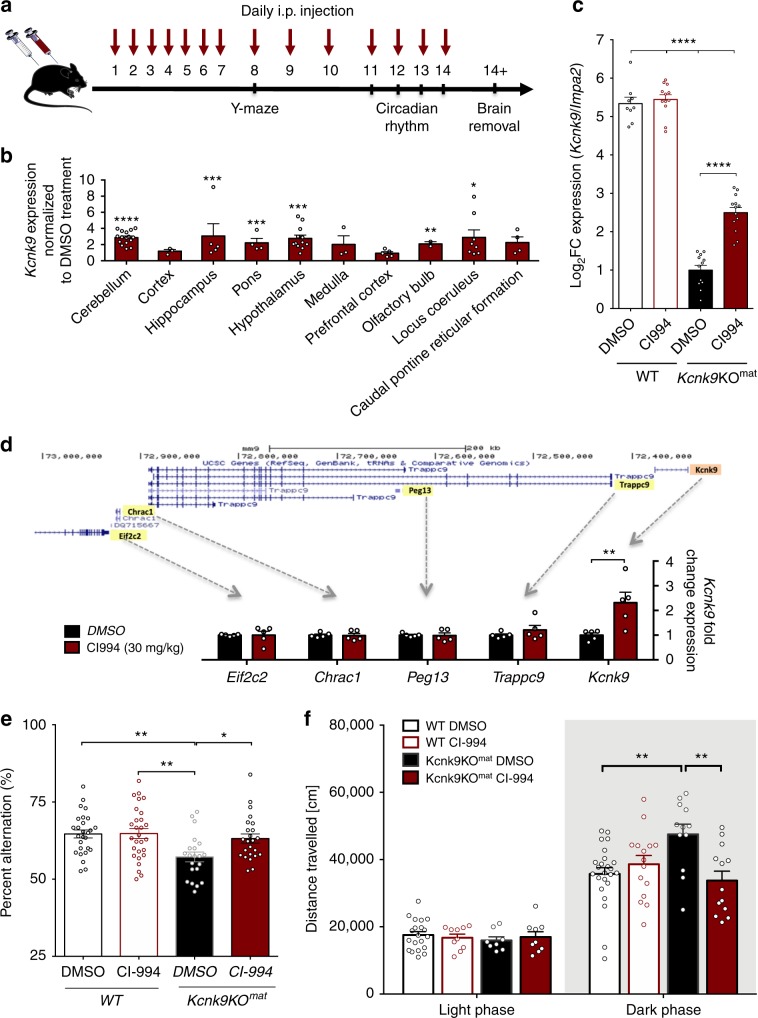
Effects of CI-994 histone deacetylase inhibitor treatment in vivo. **a** Experimental design of the mouse study. CI-994 or DMSO was intraperitoneally injected. **b** Significant upregulation of *Kcnk9* after CI-994 treatment in several brain regions normalized to DMSO treatment, Mann–Whitney U test. Regions from left to right, DMSO/CI-994, *n* = 12/13; 6/9; 12/14; 7/8; 9/12; 6/6; 9/9; 6/6; 7/8 and 7/4. **c** Upregulation of *Kcnk9* in the cerebellum of CI-994-treated *Kcnk9*KO^mat^ mice (*n* = 13), but not of CI-994-treated WT mice (*n* = 12), each compared with DMSO-treated *Kcnk9*KO^mat^ (*n* = 12) and WT mice (*n* = 9). Two-way ANOVA and subsequent Bonferroni’s multiple comparisons test, ****P < 0.0001 (all comparisons except DMSO:WT vs. CI-994:WT). **d** RT-qPCR expression analysis of known genes in the imprinting cluster on mouse chromosome 15. CI-994 treatment (30 mg CI-994/kg body weight) did not affect expression of *Trappc9, Peg13, Chrac1*, and *Eif2c2* in the hippocampus of *Kcnk9*KO^mat^ mice. Significant increase of *Kcnk9* expression after CI-994 treatment, *n* = 5, *P* = 0.0079, Mann–Whitney U test. **e** Y-maze percentage alteration was examined for DMSO-treated WT mice (*n* = 20), CI-994-treated WT mice (*n* = 18), DMSO-treated *Kcnk9*KO^mat^ mice (*n* = 21) and CI-994 treated *Kcnk9*KO^mat^ (*n* = 24) mice. CI-994 or DMSO-treated WT mice and CI-994-treated *Kcnk9*KO^mat^ mice did not exhibit deficits in working memory. CI-994-treated *Kcnk9*KO^mat^ mice showed a significant increase in percent spontaneous alteration compared with DMSO-treated *Kcnk9*KO^mat^ mice. Two-way ANOVA, Tukey post hoc test, *P < 0.05, **P < 0.01. **f** Distance traveled in the home cage in light (12 h) phase (left section) and the dark (12 h) phase (right section). No differences between DMSO-treated WT (*n* = 20), CI-994-treated WT (*n* = 10), DMSO-treated *Kcnk9*KO^mat^ (*n* = 8) and CI-994-treated *Kcnk9*KO^mat^ (*n* = 9) mice in the distance traveled in the light phase were detected. DMSO-treated *Kcnk9*KO^mat^ (*n* = 12) mice displayed a significant increase of nocturnal activity compared with DMSO-treated WT mice (*n* = 24) and CI-994-treated *Kcnk9*KO^mat^ (*n* = 13) mice as well as a visible, but not significant increase of nocturnal activity compared with CI-994-treated WT mice (*n* = 15). Two-way ANOVA, Bonferroni’s post hoc test, **P < 0.01. **b–f** *n* = sample or data arising from biologically independent animals. **e**, **f** Behavioral experiments were performed in nine independent sessions. **b–e** Values are means ± SEM (±indicates the standard error). Statistical analyses and approaches are provided in Supplementary Table 1. Source data are provided as a Source Data file. The mouse image in this figure was created using Servier Medical Art templates, which are licensed under a Creative Commons Attribution 3.0 Unported License; https://smart.servier.com.

For testing, either DMSO (100%) or CI-994 (30 mg/kg of body weight in 100% DMSO) were injected daily over 14 days in the peritoneum (Fig. 5a). After injections, *Kcnk9*^pat^ expression was induced up to ~3-fold compared with DMSO-treated controls in several brain regions including the cerebellum, hippocampus, pons, hypothalamus, olfactory bulb, and the LC (Fig. 5b). Importantly, this drug-induced rescue also included brain regions like the hippocampus, where only a small degree (<1%) of spontaneous expression from paternal alleles was observed in the *Kcnk9*KO^mat^ mice. In an allele-specific assay using WT (C57BL/6xCast/Ei)F1 hybrids, we found that CI-994 affects specifically expression of the *Kcnk9*^pat^ allele (Supplementary Fig. 4b). Only a very weak upregulation of global (maternal plus paternal) *Kcnk9* expression was seen in WT animals after CI-994 treatment (Fig. 5c, Supplementary Fig. 4c). Importantly, and in line with the in vitro observations, expression of only *Kcnk9* but no other of the genes located within the imprinting cluster on mouse chromosome 15 was found to be increased after CI-994 injection in vivo (Fig. 5d).

To assess whether CI-994-mediated enhancement of *Kcnk9* expression also promotes behavioral recovery, WT, *Kcnk9*KO^mat^, and *Kcnk9*KO^hom^ mice were subjected to behavioral testing after treatment with either DMSO (100%) or CI-994 (30 mg/kg of body weight). In the Y-maze task, CI-994 treatment led to a significant increase of spontaneous alternation in the *Kcnk9*KO^mat^ mice compared with DMSO-treated *Kcnk9*KO^mat^ control mice (Fig. 5e). Post hoc testing for multiple comparisons showed no difference between CI-994-treated *Kcnk*9KO^mat^ animals and either DMSO- or CI-994-treated WT animals (Fig. 5e). WT mice treated with either DMSO or CI-994 did not exhibit deficits in working memory and significant differences in the Y-maze. By contrast, no increase of spontaneous alternation was seen in the CI-994 treated *Kcnk9*KO^hom^ animals suggesting that the paternal allele silenced in the *Kcnk9*KO^mat^ animals carries the effect of the treatment (Supplementary Fig. 5a).

We then asked whether CI-994 treatment also alters nocturnal activity in *Kcnk9*KO^mat^ or in *Kcnk9*KO^hom^ mice. Indeed, *Kcnk9*KO^mat^ mice showed a significant decrease in horizontal nocturnal activity during the 12-h dark phase after CI-994 treatment (Fig. 5f). Again, post hoc analysis of multiple comparisons showed no significant difference between CI-994 -treated *Kcnk9*KO^mat^ and DMSO- or CI-994-treated WT animals suggesting full rescue of the nocturnal hyperactivity phenotype in the *Kcnk9*KO^mat^ animals. No drug effect was observed in *Kcnk9*KO^hom^ animals and total locomotor activity in the light phase was not altered by CI-994 treatment irrespective of the genotype of the animals (Fig. 5f, Supplementary Fig. 5b).

### CI-994 interferes with H3K27 acetylation at the *Kcnk9* locus

**Fig. 6:**
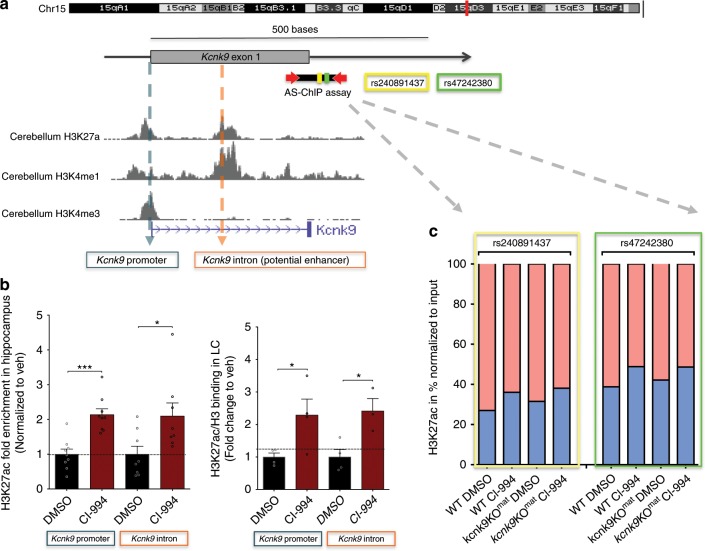
Treatment with CI-994 affects H3K27 acetylation at the *Kcnk9* locus. **a** Schematic presentation of the *Kcnk9* locus on distal mouse chromosome 15. The murine *Kcnk9* gene is shown with the corresponding H3K27ac, H3K4me1 and H3K4me3 peaks in mouse cerebellum (UCSC Genome Browser on Mouse July 2007 (NCBI37/mm9) Assembly). **b** Increased deposition of H3K27ac marks at the promoter and intronic region of *Kcnk9* in hippocampus and locus coeruleus (LC) in *Kcnk9*KO^mat^ mice following treatment with CI-994 (normalized to veh). Hippocampus (left): in *Kcnk9* promoter, DMSO (*n* = 9) vs. CI-994 (*n* = 9), *P* = 0.0001; in *Kcnk9* intron, DMSO (*n* = 8) vs. CI-994 (*n* = 8), *P* = 0.0270. LC (right) in *Kcnk9* promoter, DMSO (*n* = 4) vs. CI-994 (*n* = 4), *P* = 0.0450; in *Kcnk9* intron, DMSO (*n* = 4) vs. CI-994 (*n* = 3), *P* = 0.0202. Values are means ± SEM, *P ≤ 0.05, ***P < 0.001, by Student’s *t* test. **c** Chromatin Immunoprecipitation followed by pyrosequencing reveals allele-specific chromatin deposition of H3K27ac. Two SNPs (rs240891437 and rs47242380) in the intronic region of the *Kcnk9* in the hippocampus of (C57BL/6xCast/Ei)F1 hybrid mice were analyzed; maternal allele (red) and paternal allele (blue). Untreated state (DMSO) reveals higher acetylation levels in maternal allele compared with the paternal allele. The paternal allele exhibits higher H3K27 acetylation enrichment after CI-994 treatment compared with the maternal allele. WT:DMSO *n* = 3, WT:CI-994 *n* = 4, *Kcnk9*KO^mat^:DMSO *n* = 6, and *Kcnk9*KO^mat^:CI-994 *n* = 4. **b**, **c** *n* = biologically independent animals. Data generated in two independent experiments. Statistical analyses and approaches are provided in Supplementary Table 1. Source data are provided as a Source Data file.

Imprinting of the *Kcnk9/KCNK9* gene is thought to be regulated by a maternally methylated germline differentially methylated region (DMR) in the promoter of the *Peg13/PEG13* gene^3^. Acetylation of histone 3 lysine 27 (H3K27ac), monomethylation of histone 3 lysine 4 (H3K4me1) as well as DNA methylation at the maternally methylated DMR in the human *PEG13* promoter region have been suggested to control human *KCNK9* expression^4^ (Fig. 6a, Supplementary Fig. 6a-d). CI-994 is a second-generation class I histone deacetylase inhibitor inhibiting HDACs1 and 3 with high specificity^25^. To clarify the epigenetic mechanism underlying the induction of the *Kcnk9*^pat^ allele through CI-994 we investigated the methylation status of two subregions of the *Peg13*-DMR (*Peg13* DMR1 and *Peg13* DMR2) in adult hippocampus and LC of DMSO- and CI-994-treated *Kcnk9*KO^mat^ mice using bisulfite pyrosequencing. Methylation levels of about 40–50% typical for imprinted gene DMRs in somatic cells were determined for both DMRs in the two brain regions of *Kcnk9*KO^mat^ mice. These methylation levels were not significantly altered by CI-994 treatment of *Kcnk9*KO^mat^ mice (Supplementary Fig. 6c, d). Our results indicate that the upregulation of *Kcnk9* mRNA in various brain regions of *Kcnk9*KO^mat^ mice after CI-994 treatment was independent of the DNA methylation status at the *Peg13*-DMR.

The human *PEG13*-DMR has been further demonstrated to bind CTCF-cohesin which conveys chromatin looping between a brain-specific enhancer region, marked by H3K27ac and H3K4me1, and the *PEG13* and *KCNK9* promoters to supposedly control brain-specific *KCNK9* expression^4^. To determine the effect of CI-994 on the *Kcnk9* locus in the hippocampus and LC of *Kcnk9*KO^mat^ mice, we performed Chromatin immunoprecipitation (ChIP)-qPCR for H3K27ac and H3K4me1 marks following CI-994 or vehicle treatment. Using several public mouse brain ChIP-seq datasets, we did not find an orthologous region in the mouse displaying a brain-specific enhancer chromatin signature similar to that identified at the human imprinted domain on chromosome 8q24. Thus, we focused our analysis on the promoter and intronic region of the *Kcnk9* locus, which are enriched for both marks in the mouse brain ChIP-seq datasets (Fig. 6a). Due to the small size of the LC, we opted to use a novel low-input ChIP-method^27^ for both regions (LC and hippocampus) and validated this by conventional ChIP-qPCR in the hippocampus (Supplementary Fig. 7b). Upon CI-994 treatment, we observed a significant more than two-fold increase in H3K27ac depositions at the two *Kcnk9* regions both in the hippocampus and LC of *Kcnk9*KO^mat^ mice (Fig. 6b). The levels of H3K27ac were also increased in the hippocampus of WT littermates however the increase was not as prominent as in the *Kcnk9*KO^mat^ animals (Supplementary Fig. 7i, j). An intergenic region, lacking H3K27ac or H3K4me1 modifications, was used as negative control (Supplementary Fig. 8c-j). Looking at allele-specific H3K27ac deposition in the hippocampus of (C57BL/6xCast/Ei)F1 hybrid animals using two different SNPs in intron 1 of *Kcnk9*, we found that, as expected, the maternal allele had a higher degree of H3K27ac association than the paternal allele. CI-994 treatment of *Kcnk9*KO^mat^ mice, however, led to a higher degree of H3K27ac deposition at the paternal than at the maternal *Kcnk9* allele (Fig. 6c).

In addition, we tested the deposition of H3K4me1 both in the LC and hippocampus to assess if histone acetylation inhibition leads to additional changes in other histone marks. Treatment with CI-994 led to only a slight, but not significant increase of H3K4me1 deposition at the *Kcnk9* promoter region (Supplementary Fig. 7f-h). Our findings indicate that CI-994 treatment affects histone acetylation consistently at the promoter and intronic regions of *Kcnk9* of the investigated brain tissues and has diverging effects on H3K4me1 deposition at the investigated *Kcnk9* locus.

Taken together, our findings demonstrate that the paternal allele of *Kcnk9* is not fully silenced in the brain, further derepressed in the case of loss of the maternal allele and substantially activated upon exogenous epigenetic modulation. Our data provide evidence for a promising therapeutic effect of the class I HDAC inhibitor CI-994, in a mouse model for BBIDS with maternal loss of *Kcnk9*.

## Discussion

*Kcnk9*/*KCNK9* is an imprinted gene in mouse and man that is expressed from the maternal allele. Pathogenic variants present on only the maternal allele, therefore, cause disease in patients^1,2^. In a study comparing full *Kcnk9* knockout animals (*Kcnk9*KO^hom^) with maternal *Kcnk9* knockout animals (*Kcnk9*KO^mat^) and WT littermates in a behavioral battery, we surprisingly found an intermediate phenotype in nocturnal locomotor activity in *Kcnk9*KO^mat^ animals. Furthermore, electrophysiological analysis of tyrosine hydroxylase (TH)-positive neurons in the LC, a region involved in regulation of arousal and locomotor activity in mice, displayed significantly increased spontaneous firing frequencies only during the active dark phase in full knock-outs in contrast to WT, while it was fully rescued in the *Kcnk9*KO^mat^ animals. Allele-specific expression analysis of *Kcnk9* mRNA in (C57BL/6JxCast/Ei)F1 hybrid animals revealed significant residual paternal expression of up to 14% of total *Kcnk9* expression in the LC in the *Kcnk9*KO^mat^ animals. Daily i.p. injection of the second-generation HDAC inhibitor CI-994 could increase this expression up to three-fold and resulted in a full rescue of the behavioral phenotypes of *Kcnk9*KO^mat^ animals.

We show here that an increase in dark-phase locomotion in the *Kcnk9*KO^hom^ animals is associated with an increase of in vitro pacemaker frequency in the LC. Deletion of the active maternal *Kcnk9* allele in the mouse is supposed to lead to a phenotype comparable to the full knockout. Surprisingly, however, our study identified an intermediate behavioral phenotype in nocturnal (hyper)activity in the *Kcnk9*KO^mat^ animals, as well as a fully rescued cellular phenotype (LC firing frequency) (Figs. 1c, 3). Extended allele-specific expression analysis using (C57BL/6JxCast/Ei)F1 mouse hybrids demonstrated residual *Kcnk9* expression from the paternal allele in several brain regions with a significant peak in the LC of 14% of total *Kcnk9* expression coming from the paternal allele.

The LC plays an essential role in arousal and sleep-wake transitions^28^ and, as the origin of noradrenergic neurons projecting into the hippocampus and the prefrontal cortex, is also involved in the regulation of long-term and working memory, two essential building blocks of higher cognition/brain functions^29^. In a knockdown experiment using shRNAmir containing AAVs targeting the *Kcnk9* mRNA specifically injected into the LC we found a similar behavior phenotype in circadian activity as seen in the full and maternal *Kcnk9* knockout animals demonstrating an essential and causal contribution of *Kcnk9* expression within the LC in the control of nocturnal locomotor activity. Interestingly, our electrophysiological data show full rescue of in vitro pacemaker firing frequency through *Kcnk9*^pat^ expression in the LC in *Kcnk9*KO^mat^ animals on the one hand and only a partial rescue of dark-phase activity on the other. This might indicate different levels of *Kcnk9* expression in order to control on one hand isolated pacemaker function, which typically operates with very small ionic currents of a few picoamps, compared with in vivo function for behavioral control, where neurons are embedded in active networks and larger currents are necessary to alter their firing patterns. Our data suggest that the influence of KCNK9 on pacemaker frequency and potentially in vivo activity could be feasible as a therapeutical strategy for attention deficit and hyperactivity disorder (ADHD). However, future studies should directly record from LC neurons in vivo in awake behaving mice as well as from *Kcnk9*KO^mat^ and *Kcnk9*KO^hom^ animals to better define the functional role of KCNK9/TASK3 channels.

Nocturnal activity values (measured as distance traveled, in cm) of WT mice showed considerable variability between experiments. This variability may be attributed to various factors such as different treatments (naïve vs. injected) of the analyzed mice as well as different timepoints and environmental conditions at which the experiments were performed. It is well-known, that animal behavior heavily relies on environmental conditions within animal facilities and that these conditions can slightly change with time^30^. For example, in our animal facility, maintenance work was carried out in the time between the circadian locomotor activity experiments for the first manuscript version and those including treatment of *Kcnk9*KO^hom^ mice with CI-994 for the revised manuscript version. In light of these limitations, we strongly argue that a comparison of nocturnal activity values makes only sense within the same experiment but not across the different experiments performed.

A recent study has suggested histone methylation as a potential therapeutic target for another imprinting disorder, the Prader-Willi syndrome (PWS)^31^. The authors showed that UNC0642, a selective inhibitor of euchromatic histone-lysine N-methyltransferase-2, derepressed the maternal copies of paternally expressed PWS candidate genes and promoted the growth and survival of mouse pups with a paternal deletion of the PWS region without altering DNA methylation at the PWS-ICR. We show here that derepression of the paternal allele in *Kcnk9*KO^mat^ animals leads to a significant increase in *Kcnk9* expression in selected brain regions and that this is enough to substantially influence the behavioral phenotype in domains likely to be regulated by LC activity. Furthermore, we found that the second-generation HDAC inhibitor CI-994 is another attractive option for treatment of imprinting disorders such as BBIDS. CI-994 treatment of *Kcnk9*KO^mat^ mice as a mouse model of BBIDS substantially activated the repressed paternal allele of *Kcnk9* in several brain areas resulting in a significant increase in *Kcnk9* expression. This led to the full rescue of specific domains of brain function impaired in BBIDS. Our data for the first time shows successful phenotypic rescue of impaired brain function in a mouse model for an imprinting disorder using an epigenetic modulator.

Similar to the findings in the UNC6042-treated mouse model of PWS^31^, activation of the *Kcnk9*^pat^ allele was not accompanied by changes of DNA methylation at the *Peg13* DMR, which is assumed to play a key role in regulation of *Kcnk9* imprinting also in the mouse^3^. Notably, the bisulfite technique did not allow us to discriminate between 5-methylcytosine and 5-hydroxymethylcytosine levels possibly affected by CI-994. Though, our data strongly argue that CI-994-mediated derepression of the *Kcnk9*^pat^ allele is primarily caused by an increase of H3K27ac at regulatory elements in the promotor and intronic region of the *Kcnk9* gene. However, the treatment did not display significant differences in H3K27ac deposition between the LC and hippocampus tissues suggesting a region-independent effect of the HDAC inhibitor. Such effects have commonly been reported in multiple genome-wide studies investigating the effect of HDAC inhibitors^32^. Interestingly, CI-994 as a histone deacetylase inhibitor did not only selectively increase H3K27ac but also slightly increased H3K4me1 at the *Kcnk9* promoter region. This is not unexpected since crosstalk mechanisms between histone-modifying enzymes often affect the binding and activity of further histone-modifying enzymes. For instance, treatments with HDAC inhibitors were reported to affect globally the histone methylation marks specifically of H3K4me1^32^. These observations are repeatedly described in the literature suggesting a broad effect of HDAC inhibitors on chromatin structure and transcription^33,34^.

In general, epigenetic modulation is supposed to rather unspecifically target numerous genes and their expression in the genome. Side effects of all sorts including tumor development and metabolic dysregulation are believed to be likely with such substances. Also, interference with brain function leading to cognitive impairment and mental dysfunction cannot be excluded. However, valproic acid, a first generation HDAC inhibitor has been used in anti-epileptic therapy for decades without severe tumorigenic or metabolic long-term effects. CI-994 is a second-generation, specific class I HDAC inhibitor. Although a relatively broad effect of CI-994 on gene expression has been demonstrated in vivo, it is being used in several clinical trials for anti-cancer therapy having proven inhibitory potency of epithelial-mesenchymal transition processes^35^. Accumulating evidence also suggests that CI-994 provides neuroprotective effects in the central nervous system and cell survival in vitro^36^. Regarding brain function in WT mice no effect of CI-994 treatment has been seen in the open field^26^. In our experiments, no significant influence of two weeks CI-994 injection was seen on working memory or circadian locomotor activity in WT animals. Furthermore, an increase of *Kcnk9*^pat^ expression after CI-994 injections was only seen in selected brain regions, but not in, e.g., cortex or prefrontal cortex, and mainly in the maternal knockout animals (only very weakly in WT animals). Allele-specific ChIP-qPCR of (C57BL/6JxCast/Ei)F1 hybrids showed that the CI-994 effect is more pronounced on the paternal allele (Fig. 6c). This leads to a visible increase in expression of the paternal but not the maternal allele (Supplementary Fig. 4a) and might be due to the high acetylation status of the active maternal allele. Furthermore, CI-994 treatment did not affect the expression of other nearby imprinted genes within the imprinted cluster on mouse chromosome 15 in vitro and in vivo. Taken together these data suggest a rather specific epigenetic action of CI-994 in *Kcnk9*KO^mat^ animals, which might make CI-994 safe for usage in BBIDS patients. However, so far all CI-994 related studies have been carried out in vitro or in adult animals. If used in patients, early treatment during development might be more efficient. In order to make this possible, further studies on CI-994 specificity and possible adverse and teratogenic effects in young mice have to be conducted.

In summary, we show here that brain region-specific upregulation of the paternally silenced *Kcnk9* allele particularly in the LC of *Kcnk9*KO^mat^ animals modulates nocturnal hyperactivity, a central phenotype of *Kcnk9* knockout animals. We further show that the class I HDAC inhibitor CI-994 leads to a significant additional increase of *Kcnk9*^pat^ expression in several brain regions and fully rescues behavioral alterations in *Kcnk9*KO^mat^ animals. This rescue is associated with an increase of H3K27 histone acetylation at the promotor and intronic region of the *Kcnk9* gene but not with changes in DNA methylation at the differentially methylated region in the *Peg13/Kcnk9* locus. Our data suggest epigenetic modulation by CI-994 as a promising therapeutic strategy in patients with BBIDS and, more generally, derepression of imprinted gene alleles as a sustainable approach for the treatment of imprinting disorders.

## Methods

### Mice

All experimental procedures were performed in accordance with institutional animal welfare guidelines and were approved by the ethical committee of the state government of Rhineland-Palatinate, Germany (ID: 23 177-07/G 17-1-022). *Kcnk9*KO^hom^ mice were provided by Florian Lesage, Institut de Pharmacologie Moléculaire et Cellulaire, Valbonne, France. The gene targeting strategy of *Kcnk9*KO^hom^ mice was based on a cre-mediated deletion of exon 2 encoding pore domains P1 and P2, transmembrane domains M2–M4 as well as the cytoplasmic C-terminus^18^. Breeding of two heterozygous animals revealed homozygous, heterozygous and WT animals at a proportion of 1:2:1. WT littermates were used as controls. WT and heterozygous *Kcnk9*KO mice with inactivation of the maternal *Kcnk9* allele (*Kcnk9*KO^mat^) were obtained by crossing male WT mice with female *Kcnk9*KO^mat^ mice. Finally, breeding of homozygous female and heterozygous male mice resulted in *Kcnk9*KO^mat^ and *Kcnk9*KO^hom^ mice. The mice were kept under specific-pathogen-free (SPF) conditions on a 12 h light/12 h darkness cycle in standard polystyrene cages with free access to water and food. Using tail tip DNA, mice were genotyped by assessing exon 2 excision using the primers flanking this region (F, 5′-TGCGAGCTTCAGAGAGAGGATG-3′ and R, 5′-ATGCTCTAATCTCCAGTCTG-3′) producing fragments for WT and mutant alleles. Additional primers within exon 2 were applied to generate a control product for the WT allele (F, 5′-CACCACGCCATGTACTTCCT-3′ and R, 5′-GGACCGGAAGTAGGTGTTCC-3′). Male mice at 8–10 weeks of age were used for expression analysis and to investigate the behavioral phenotype with and without drug treatment. Allele-specific expression analyses were carried out with total RNA from F1 offspring derived from crosses between female WT C57BL/6J or *Kcnk9*KO^hom^ mice and male WT Mus musculus castaneus (Cast/Ei) mice.

### Behavioral testing

Littermate WT and *Kcnk9*KO mice were tested in 4–8 cohorts of mice, with testing beginning at 8–10 weeks of age. All experimenters were blinded to the genotype of the mice throughout the studies and behavioral analyses. Immediately after the behavioral tests, animals were sacrificed and whole brains were rapidly removed and incubated in RNAlater® (Sigma) for subsequent expression analysis.

### Drug administration in vivo

Nine weeks old male *Kcnk9*KO^mat^ and WT mice were injected intraperitoneally once daily with CI-994 (ApexBio) or dimethyl sulfoxide (DMSO) (control vehicle) for 7 consecutive days before behavior experiments were initiated (Fig. 5a). On days of testing, mice were injected 2 h before behavior experiments. A single injection contained either 20 µl DMSO or 20 µl CI-994 (35 mg/kg) dissolved in 100 % DMSO.

### Toxicity/viability test in CI-994 treated mPCNs

mPCNs were washed with PBS and fixed with 4% paraformaldehyde for 2 h. Subsequently, cells were incubated for 20 min in 0.4% Triton X-100 in PBS at 37 °C and further rinsed three times with PBS. After blocking with 10% sheep serum at 37 °C for 1 h, cells were washed for a further three times with PBS. Next, neurons were incubated overnight at 4 °C with anti-neuron-specific nuclear protein NeuN monoclonal antibody (cat. # ab104225; dilution 1:200 in PBS), followed by five washes with PBS for 5 min each. The neurons were subsequently incubated with an Alexa Fluor 488-conjugated goat anti-mouse secondary antibody (cat # ab150077; dilution 1:200 in PBS) for 1 h at 37 °C and washed for a further five times in PBS. After staining, cells on coverslips were mounted onto microscope slides and counterstained with mounting medium containing 2 μg/mL Hoechst 33258 (Cat # H3569).

### Tissue collection

Whole mouse brains were removed and incubated in RNAlater® (Sigma) and stored for 2 days at 4 °C. After the dehydration process, several brain regions were dissected and further processed or stored at −80 °C. For LC samples, coronal brain sections (80 μm) between bregma −5.40 and −5.52^37^ were generated, and 0.51 µm punches bilaterally of each brain region were collected with the Brain Punch Tissue Set (Leica). The accuracy of the LC tissue collection was validated by performing tyrosine hydroxylase (TH) expression analyses and comparing the expression levels to those of WT cerebellum and hippocampus samples. Samples with non-adequate TH levels were excluded from analysis. Tyrosine hydroxylase, which serves as a norepinephrine marker, was expressed ~400-fold higher compared with hippocampus samples, indicating a precise tissue dissection (Supplementary Fig. 2).

### Cell culture

*Kcnk9*KO^mat^ primary cortical neurons (mPCNs) were isolated from E14 embryos derived from matings between female *Kcnk9*KO^hom^ mice and WT males. After collection of brains, cortices were dissected out and mechanically separated into single cortical cells through resuspension. The mPCNs were plated on Poly-L-Ornithine (Sigma)- and Laminin (Sigma)-coated plates and maintained in culture medium containing Neurobasal medium (Gibco), supplemented with 2% B27 plus vitamin A (Gibco) and 1% Glutamax (Gibco), in a humidified incubator at 37 °C and 5% CO_2_.

### In vitro drug treatment

We cultured murine primary cortical neurons (mPCNs) for 4 days in 6- or 12 well-plates and treated them with epigenetic compounds diluted in culture medium for 24 or 72 h. To investigate the duration of *Kcnk9* unsilencing, mPCNs were cultured for 3 days and treated with 20 μM CI-994. After 2 days of treatment, cells were washed with 37 °C culture medium. After 1 day, some cells were harvested. Medium change was conducted after additional three days. Six days later, on 10 days, remaining cells were harvested and analyzed via RT-qPCR.

### AAV generation and *Kcnk9* knockdown in WT mice

Viral constructs were purchased from Sirion Biotech (Martinsried, Germany). Generation and evaluation of shRNAmir sequence targeting the gene *Kcnk9* were performed using SIRION’s RNAiONE platform as previously described^38^. Ten different 21mer shRNAmir sequences against *Kcnk9* were selected using algorithms listed in Fig. 2a. Corresponding shRNA template oligonucleotide cassettes were cloned into a shuttle plasmid under the control of the human U6 promoter. The coding region of the cDNA was PCR-amplified and cloned into the validation vector pVal downstream of the EGFP coding region resulting in pVal-target. The promoter-shRNA regions of the shRNA shuttle plasmids along with a shuttle plasmid encoding for a NT-shRNA as control were then transferred into pVal-target by recombinational cloning. Cells were then transfected with the validation plasmids and were incubated for 48 h. Total RNA was subsequently isolated and 1 μg was reverse transcribed using a mixture of random hexamer and oligo-dT primer. The silencing efficiency of each shRNA was determined by quantification of the EGFP-target cDNA expression levels relative to NT-shRNA control vector. The expression cassette was then cloned into an AAV transfer vector (pAAV-Syn-shRNAmir-*Kcnk9*-EF1a-eGFP and pAAV-Syn-shRNAmir-scrambled-EF1a-eGFP) and ITRs integrity was then validated by restriction analysis. 293T cells were utilized for AAV production, which were generated via co-transfection and transferred to 1E13 VG in vivo buffer. We infused 8-week-old C57BL/6J mice (CLR) with either AAV1-hSyn-shRNAmir-*Kcnk9*-EF1a-eGFP (Sirion, Germany) to knockdown the *Kcnk9* expression or AAV1-hSyn-shRNAmir-scrambled-*Kcnk9*-EF1a-eGFP (Sirion, Germany) as a control bilaterally in the LC. Animals where anesthetized with isoflurane (1–2%) and received a subcutaneous injection of carprofen (4 mg/kg). Three hundred nanoliters of virus was infused with a rate of 100 nl/min in the LC (AP: −5.4, ML: ±1.0, DV: 3.0, in mm, relative to bregma) under stereotaxic control (Kopf Instruments, USA) using a 1 µl Hamilton syringe with a glass capillary. Twenty-one days after surgery spontaneous alteration and circadian rhythm were tested.

### RNA Isolation, cDNA synthesis, and RT-qPCR

The total RNA extraction with TRIzol reagent from brain tissue was prepared as suggested by Invitrogen Life Technologies. High Pure RNA Tissue Kit (Roche) and High Pure RNA Isolation Kit (Roche) were applied to extract total RNA from small tissue and cell samples using spin columns. The purity, quantity, and integrity of the RNA were measured with a NanoDrop 1000 spectrophotometer and an Agilent 2100 Bioanalyzer. The cDNA samples were synthesized from 200 to 1000 ng total RNA using the PrimeScript^TM^ RT Master Mix cDNA (Takara) as recommended by the manufacturer. Quantitative real-time PCR (qRT-PCR) was carried out using SYBR® Premix Ex Taq™ II (Tli RNaseH Plus) and 10 μM primers (final concentration), according to the manufacturer’s instructions. Allele-specific RT-qPCR reactions were performed on an ABI StepOnePlus^TM^ Real-Time PCR System using exon–exon junction primers with the following conditions: 95 °C/30 s, 40 cycles of 95 °C/5 s, 68.5 °C/30 s, 72 °C/30 s. All additional RT-qPCR reactions were performed with the following conditions: 95 °C/30 s, 40 cycles of 95 °C/5 s, 60 °C/30 s, 72 °C/30 s. All reactions were measured in triplicates, and median cycles to threshold (Ct) values were used for analysis. The housekeeping gene *Impa2* was used to normalize against experimental genes, and relative gene expression was determined using the 2−ΔΔCT methods^39^. *Impa2* forward primer 5′-CGTGCGGGACAAATCATCAG-3′ and reverse primer 5′-CGTGCGGGACAAATCATCAG-3′.

### Allele-specific expression analysis by QUASEP

For allele-specific expression analysis, the synthesized cDNA of (C57BL/6JxCast/Ei)F1 hybrid mice was amplified in a PCR applying one non-biotinylated forward primer: 5′-GCCTGTACCTTCACCTAC-3′ and one biotinylated reverse primer: 5′-CACAACTATCGGATATGGACATGC-3′. To distinguish the origin of the alleles we subsequently analyzed the RT-PCR products in allele-specific expression analysis, using the C/T-SNP rs225149059 (Ensembl; GRCm38.p5) and QUASEP method as previously described^3^. A *Kcnk9*-specific pyrosequencing primer: 5′-TGCCGCGGTGTTC-3′, was designed flanking the allele-specific SNP and pyrosequencing was carried out on a PyroMark Q96 instrument (Qiagen).

### DNA methylation analysis

Approximately 500 ng DNA was subjected to sodium bisulfite treatment and purified using the EZ DNA Methylation Direct™ kit (Zymo Research). Bisulphite PCR primers for the *Peg13*-DMR1 and *Peg13-*DMR2 (located from +195 to +833 bp relative to the 5′ end of the *Peg13* transcript) were designed using the Pyrosequencing Assay Design Software (Qiagen). PCR amplification was performed with 1 μg bisulfite converted DNA and specific primers at 42 cycles in a bisulfite PCR analysis. We amplified two CpG-rich regions, *Peg13*-DMR1 and *Peg13*-DMR2, associated with the mouse *Kcnk9* gene regulation with the following primers: *Peg13*-DMR1, forward 5′-TTGGATGAGTTATTATATAAGGTTTAAAA-3′ and reverse 5′-ACAACTACCTACATTCCAAATCT-3′-biotinylated (product size: 175 bp), sequencing 5′-AAATTTTAATAAGATGGGTTAAT-3′. *Peg13*-DMR2, forward 5′-AGATTTGGAATGTAGGTAGTTGTGA-3′ and reverse 5′-CCTCAATAAAACCATTCTAATCAACTAT-3′-biotinylated (product size: 198 bp), sequencing 5′-GGTAATTTGTTAGGTGGAGATATA-3′. The pyrosequencing was carried out on the PyroMark Q96 instrument (Qiagen).

### Chromatin Immunoprecipitation (ChIP)-qPCR

ChIP experiments were performed as described in Akhtar et al. with minor modifications for brain tissue processing^27^. Briefly, brain tissues were dissected in cold PBS and homogenized with a pestle. Following dissection, tissues were fixed with 1% formaldehyde in PBS for 10 min at room temperature, followed by quenching of the fix with 125 mM glycine for 5 min. Fixed cells were resuspended in 140 mM RIPA (10 mM Tris-Cl pH 8.0, 140 mM NaCl, 0.1 mM EDTA pH 8.0, 1% Triton X-100, and 0.1% SDS) and subjected to 18 cycles of sonication on Bioruptor Pico (Diagenode), with 30 secs “ON”/ “OFF” at high settings. After sonication, samples were centrifuged at 14,000 × *g* for 10 min at 4 °C and supernatant was transferred to a fresh tube. The extracts were incubated overnight with 1 µg (1:500 dilution) of H3K27ac (Abcam, Cat # ab4729), H3K4me1 (Active motif, Cat # 39297), H3 (Abcam, Cat # ab1791) or IgG control (Diagenode, Cat # C15410206) antibody at 4 °C with head-over tail rotations. After overnight incubations, 20 µl of blocked Protein A and G Dynabeads (Diagenode) were added to the tubes and further incubated for 3 h to capture the antibodies. Bead separation was carried out using a magnetic rack and washed as following; once with 140 mM RIPA (10 mM Tris-Cl pH 8.0, 140 mM NaCl, 0.1 mM EDTA pH 8.0, 1% Triton X-100, and 0.1% SDS), four times with 250 mM RIPA (10 mM Tris-Cl pH 8.0, 250 mM NaCl, 0.1 mM EDTA pH 8.0, 1% Triton X-100, and 0.1% SDS) and twice with TE buffer pH 8.0 (10 mM Tris-Cl pH 8.0 and 0.1 mM EDTA pH 8.0). For reversal of cross-linking, samples were RNase-treated (NEB) and subjected to Proteinase K treatment for 12 h at 37 °C and at least 6 h at 65 °C after the immunoprecipitation. After proteinase K treatment, the DNA was extracted using the phenol-chloroform method. After precipitating and pelleting, DNA was dissolved in 30 µl of TE buffer pH 8. The real‐time PCR was performed using SYBR Green chemistry (ABI) on the ABI StepOnePlusTM Real-Time PCR System. The input and chromatin immunoprecipitated material were processed identically across the samples. The fluorescent signal of the amplified DNA was normalized to H3 binding or input with the following primers: *Kcnk9* promoter F 5′-CGTGTGCGCTACATCTCCTA-3′ and R 5′-ATTCGCCGGTTCCTTCTACT-3′; *Kcnk9* intron F 5′-AGGGCAGATGCTTAAGAGGA-3 and R 5′-CATCTGTTCTGTACCCCATCC-3′; *Intergenic region 3 (Ig3)* F 5′-ATGCCCCTCAGCTATCACAC-3′ and R 5′-GGACAGACATCTGCCAAGGT-3′. The low amount chromatin immunoprecipitation (TAF-ChIP) was performed according to Akhtar et al.^27^.

### Allele-specific ChIP followed by pyrosequencing

For allele-specific ChIP analysis, DNA of WT and *Kcnk9*KO^mat^ (C57BL/6JxCast/Ei)F1 hybrid mice, treated either with DMSO or CI-994, was amplified in a PCR applying one non-biotinylated forward primer: 5′-AAATTCGCCGGTTCCTTCTAC-3′ and one biotinylated reverse primer: 5′-GAGATGTAGCGCACACGAAGC-3′. To distinguish the origin of the alleles we subsequently analyzed the PCR products in an allele-specific manner, using two neighboring SNPs, rs240891437 and rs47242380 (Ensembl; GRCm38.p5). The quantification of enrichment was performed using the pyrosequencing technique. A *Kcnk9*-specific pyrosequencing primer: 5′-AAGGAATGGGTGTGC-3′ was designed and pyrosequencing was carried out on a PyroMark Q96 instrument (Qiagen).

### Behavioral analyses

Brief descriptions of the behavioral tests are provided below. Male mice between 8 and 10 weeks of age were used for behavioral tests, as described below.

### Circadian locomotor activity

Mice were individually placed in transparent polypropylene cages (38 × 22 × 15 cm) with standard beddings. Food and water were available and accessible at all times. The cages were placed in a sound-prove room under dimed light during the light phase and under infrared LED lights during the dark cycle. The horizontal locomotor activity of the mice was recorded in the light and in the dark phase using a video tracking software (EthovisionXT, Noldus software). The circadian rhythm activity was measured after 48 h acclimatization for 24 h and analyzed with EthovisionXT, Noldus software. The results show the distance traveled (in cm) in the light and in the dark phase.

### Spontaneous alternation

Spatial working memory was assessed in a Y-maze test as described previously^40^. During a 10-min session in a Y-maze the spontaneous alternation behavior was recorded. The spontaneous alternation test is based on the willingness of rodents to explore new environments, therefore to enter the arm of a Y-maze that had not been recently explored, i.e., the arm that was not entered in the previous choice. Mice were placed in a gray plexiglass Y maze in a sound proof room. Each maze consisted of three arms. Each arm was 40-cm long, 20-cm high, and 10-cm wide, and the arms converged to an equilateral triangular central area. Each mouse was placed at the end of the start arm, in which the mouse starts to explore and move freely through the maze during the 10‐min session. The series and order of arm entries was observed manually and considered to be completed when the hind paws of the mouse had completely entered the arm. Transitions between arms were scored as either correct or incorrect alternations by the observer. Healthy animals (controls) show a high change rate. This shows that the animal can remember which arm was last entered. The percentage of alternation was calculated as the ratio of actual to possible alternations multiplied by 100%, either for a 10 min session or 25 initial decisions. Each of the arms of the Y maze was cleaned with 70% ethanol solution between trials.

### In vitro brain slice patch-clamp recordings of LC neurons

Adult mice were anesthetized and perfused with ice-cold ACSF as previously described^41^. The brainstem slices containing the LC were sectioned after intracardial perfusion using ice-cold ACSF (50 mM sucrose, 125 mM NaCl, 2.5 mM KCl, 25 mM NaHCO_3_, 1.25 mM NaH_2_PO_4_, 2.5 mM glucose, 6.2 mM MgCl_2_, 0.1 mM CaCl_2_, and 2.96 mM kynurenic acid; Sigma-Aldrich GmbH), oxygenated with 95% O_2_/5% CO_2_). Slices were continuously perfused with oxygenated ACSF (2–4 ml min^−1^, 36 °C; 22.5 mM sucrose, 125 mM NaCl, 3.5 mM KCl, 25 mM NaHCO_3_, 1.25 mM NaH_2_PO_4_, 2.5 mM glucose, 2 mM MgCl_2_, and 2 mM CaCl_2_; 95% O_2_/5% CO_2_) for ≥45 min and then transferred to a recording chamber. CNQX (12.5 μM; Biotrend), AP-5 (10 µM; Biotrend), gabazine (SR95531, 4 μM; Biotrend), and yohimbine (1 µM; Biotrend) were added to inhibit fast excitatory and inhibitory synaptic transmission as well as activity of somatodendritic alpha2 adrenoreceptors, respectively. The electrophysiological recordings (whole-cell patch-clamp recordings) and data acquisition were performed as previously described^42^.

### Histology

Animals were injected with phenobarbital and perfused with fixation solution containing 4% paraformaldehyde, 15% picric acid in phosphate buffer solution (PBS). The removed brains were post-fixed overnight, 60 µm brain sections were sliced using a microtome (VT1000S, Leica). Free-floating sections were washed in PBS and incubated with blocking solution (10% horse serum, 0.5% Triton X-100% and 0.2% BSA in PBS) for 1 h at room temperature. Then, sections were incubated with primary antibodies in carrier solution (1% horse serum, 0.5% Triton X-100% and 0.2% BSA in PBS) at room temperature overnight. After washing the sections with PBS, they were incubated with secondary antibodies in carrier solution overnight. The following primary antibodies were used: monoclonal mouse anti-tyrosine hydroxylase (TH, catalog #MAB318, 1:1000, Millipore), polyclonal rabbit anti-GFP (catalog #A11122, 1:1000, Life Technology). The following secondary antibodies were used: Alexa Fluor 488 goat anti-rabbit (catalog #A11008, 1:1000, Thermo Fisher Scientific, Invitrogen) and Alexa Fluor 568 goat anti-mouse (catalog #A11004, Thermo Fisher Scientific, Invitrogen). On the third day, sections were washed with PBS, incubated in PBS containing 0.02% 4′,6′-diamidin-2-phenylindol (DAPI, catalog #D1306, Molecular Probes, Invitrogen) for 5 min and washed in PBS for 10 min. Afterward, sections where mounted on slides, coverslipped and stored at 4 °C until confocal images were taken.

### Statistical analyses and graphical illustrations

All analyses and graphical illustrations were performed using Prism 7 (Graphpad Software, San Diego, CA) Assumptions concerning normal distribution were applied (Kolmogorov–Smirnov test) before statistical analyses were implemented. Statistical analyses and approaches are provided in Supplementary Table 1.

Concerning annotation, the sample/cell number is expressed with n and number of mice is noted with N. For animal studies and RT-qPCR expression analysis, data were compared using a one-way ANOVA and two-way ANOVA with and without repeated measures by multiple comparisons followed by Tukey’s or Bonferroni’s multiple comparison post hoc test. The numbers of biologically independent experiments, sample size, statistical results and ages of the animals are all indicated in the main text or figure legends and Supplementary Table 1. Values were only excluded if they were identified as outliers based on a Grubbs’ test. Unless stated otherwise, the data given in figures and text are expressed as mean ± SEM. *P* < 0.05 was considered to be statistically significant.

### Reporting summary

Further information on research design is available in the Nature Research Reporting Summary linked to this article.

## Supplementary information

Supplementary Information

Reporting Summary

## Data Availability

All relevant data are available from the authors. The source data underlying all figures and supplementary figures are provided as a Source Data file.

## Source Data

Source Data
