# Supplementary material for: Inhibition of histone deacetylation rescues phenotype in a mouse model of Birk-Barel intellectual disability syndrome
Source: Nat Commun. 2020 Jan 24;11:480. doi: 10.1038/s41467-019-13918-4 (PMC6981138; doi:10.1038/s41467-019-13918-4)
Supplement: Supplementary file 1 — Supplementary Information [file 41467_2019_13918_MOESM1_ESM.pdf]

Supplementary Information

Inhibition of histone deacetylation rescues phenotype in a mouse model of Birk-Barel Intellectual Disability syndrome

Cooper et al.

Supplementary Figures

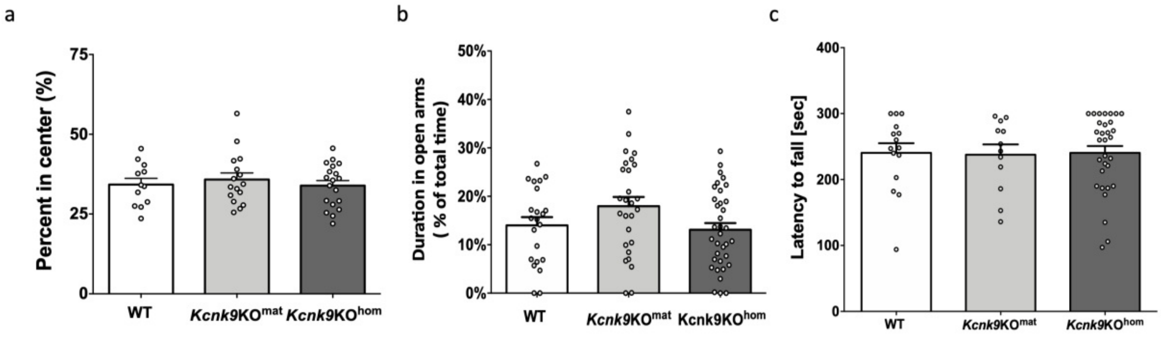

Supplementary Figure 1: Deletion of *Kcnk9* does not affect anxiety and motor coordination of *Kcnk9*KO mice. No significant differences were observed in **(a)** the open-field-test between WT (n=12), *Kcnk9*KO^{mat} (n=16) and *Kcnk9*KO^{hom} (n=19) mice, **(b)** the elevated-plus-maze between WT (n=22), *Kcnk9*KO^{mat} (n=27) and *Kcnk9*KO^{hom} (n=35) mice, and **(c)** the rotarod-test between WT (n=15), *Kcnk9*KO^{mat} (n=12) and *Kcnk9*KO^{hom} (n=31) mice using One-way ANOVA and followed Bonferroni's multiple comparison post hoc test. Values are means ± SEM. **(a-c)** Behavioral data are from biologically independent animals (n= number of mice). Statistical analyses and approaches are provided in Supplementary Table 1. Source data are provided as a Source Data file.

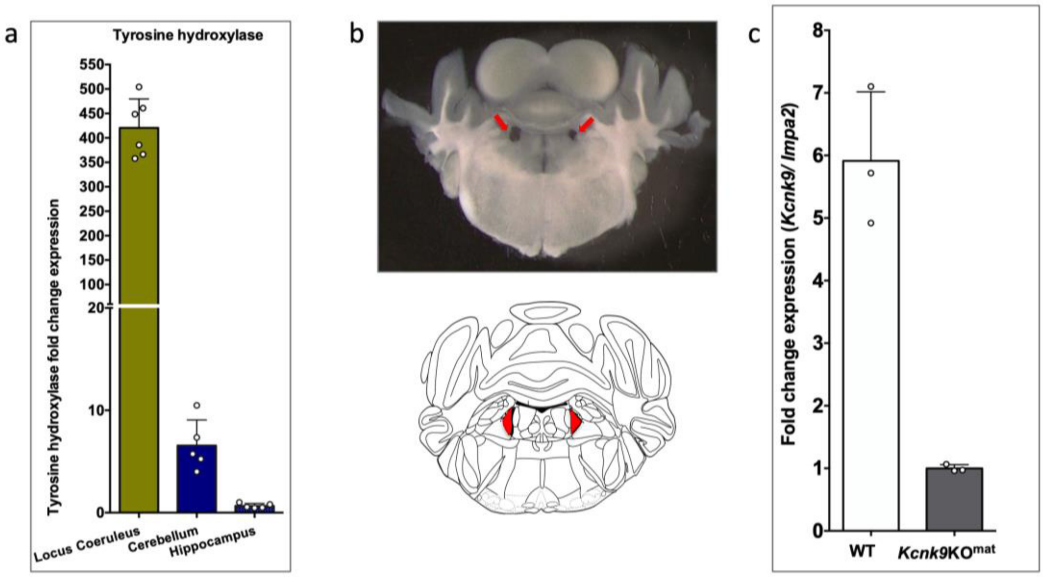

Supplementary Figure 2: Locus coeruleus expression analysis (a) Tyrosine hydroxylase (TH) RT-qPCR expression analysis. The TH expression was highly increased in locus coeruleus (LC, n=6) compared to cerebellum (n=5) and hippocampus (n=5) samples of WT mice. TH serves as a norepinephrine marker. **(b)** Coronal brain slice of an adult mouse; red arrows indicate tissue excision position of LC (top) schematic coronal section of the mouse brain at the position -5.4 relative to bregma[1]; LC is depicted as a red triangle (bottom) **(c)** *Kcnk9* RT-qPCR expression analysis in LC samples of WT (n=3) compared to those of *Kcnk9*KO^{mat} (n=3) mice. **(a,c)** n= biologically independent samples from individual mice. Values are means ± SEM. Source data are provided as a Source Data file.

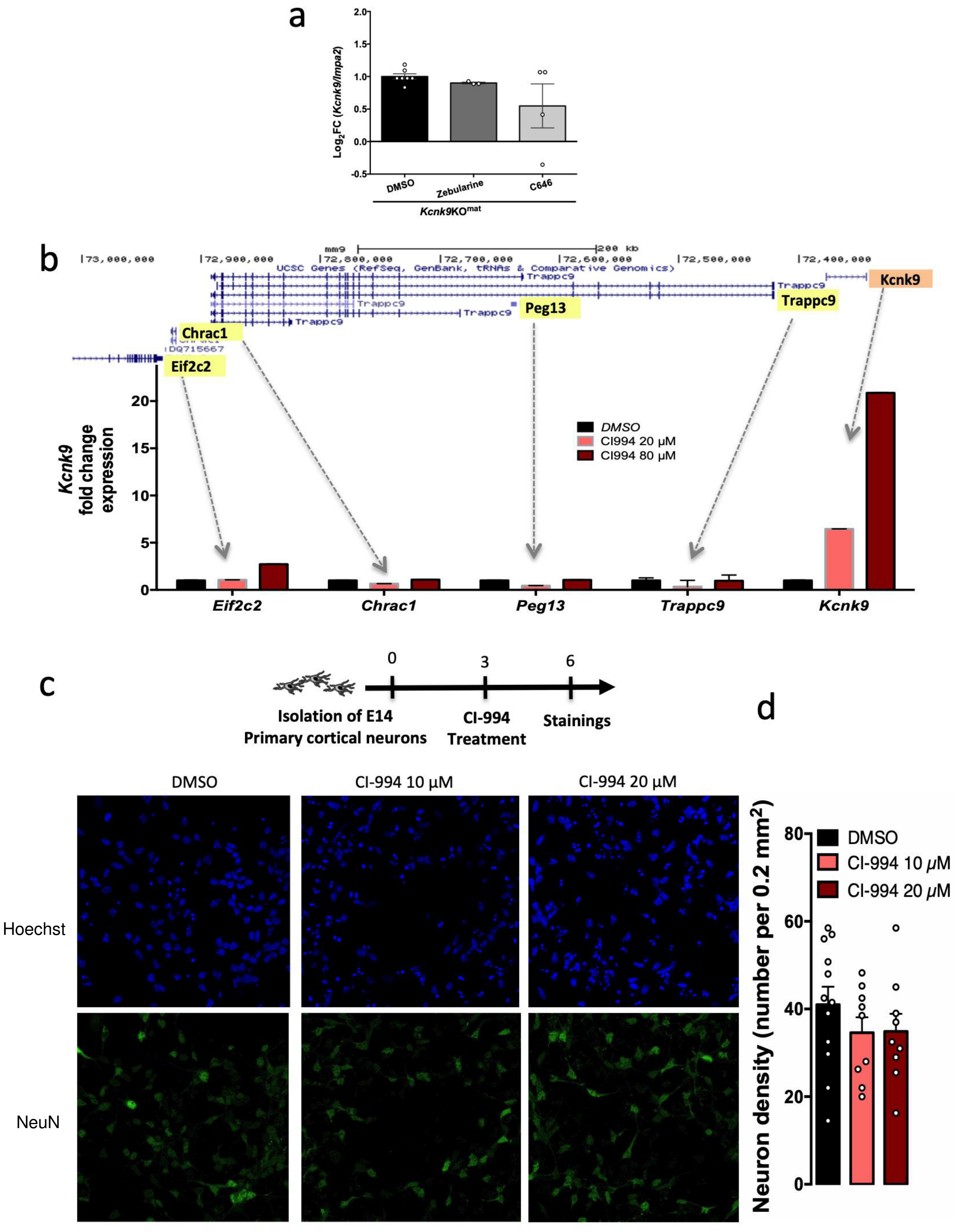

Supplementary Figure 3: Epigenetic drug treatments in murine primary cortical neurons (mPCNs). (a) The compounds Zebularine and C646 show no effect on the *Kcnk9* expression in *Kcnk9*KO[mat] mPCNs (Zebularine P=0.0667, C646 P=0.4727, Mann-Whitney-U test. **(b)** RT-qPCR expression analysis of known genes in the imprinting cluster on mouse chromosome 15. CI-994 treatment (20 µM and 80 µM CI-994) did not affect expression of *Trappc9, Peg13, Chrac1* and *Eif2c2* in mPCNs (n=2-3 cultures/group). **(c)** Evaluation of toxicity/viability in CI-994 treated mPCN compared to DMSO-treated controls. Cells were stained with Hoechst 33258 (nucleus) and NeuN (mature neurons) 3 days after treatment with either DMSO or CI-994. **(d)** Neuron density after CI-994 treatment. No significant difference in neuronal density was observed between DMSO (n=12), 10 µM CI-994 (n=9) and 20µM CI-994 (n=9) treated mPCNs. Values are means ± SEM. Statistical analyses and approaches are provided in Supplementary Table 1. Source data are provided as a Source Data file.

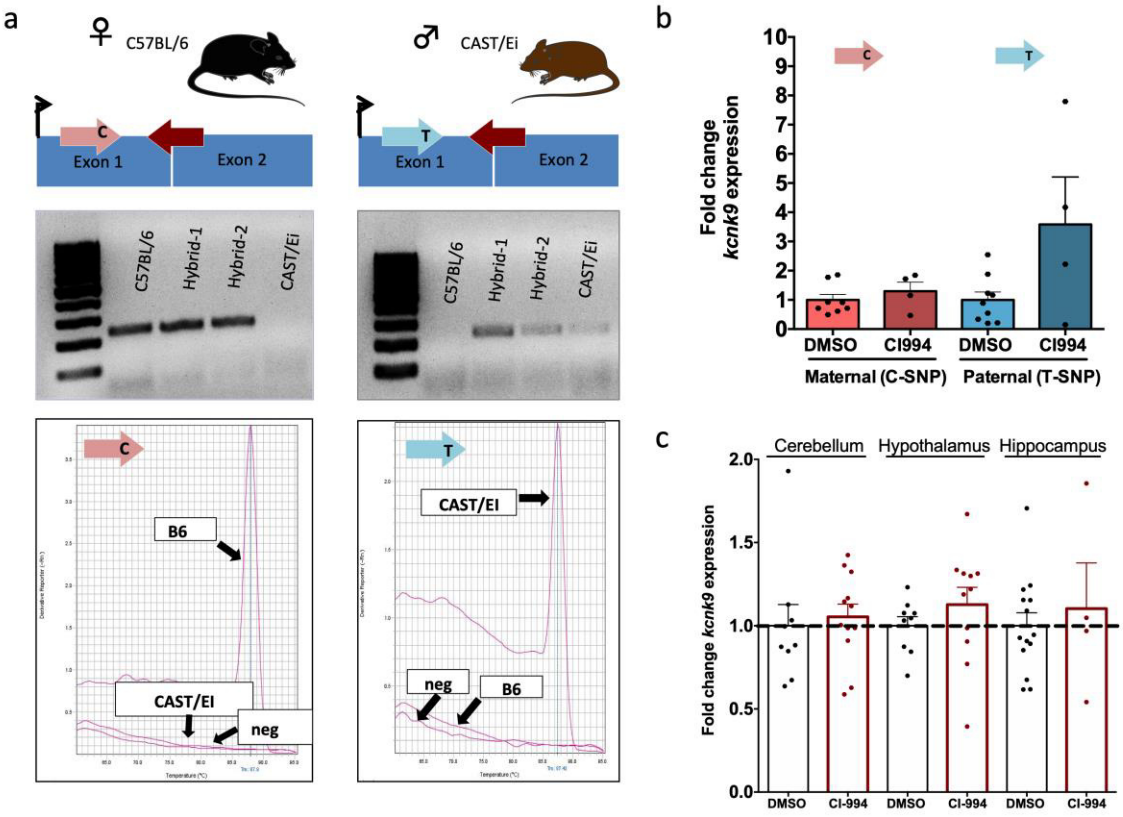

Supplementary Figure 4: Allele-specific de-repression of *Kcnk9* in the mouse brain (a) Assay design and melt curve analysis for the allele-specific RT-qPCR (AS-RT-qPCR) of *Kcnk9*. Allele-specific primers bind to the strain-specific SNP at the 3´-end, allowing only the complementary primer to elongate (top). Agarose gel electrophoresis of PCR product (231 bp) loaded on 2% agarose gel using a 100 bp DNA ladder (middle). Melt curve analysis shows a primer-specific binding (bottom). **(b)** Analysis of expression levels using the C57BL/6 C and Cast/Ei T allele-specific primer and normalization with a reference gene revealed an increased expression of the paternal *Kcnk9* allele in the hippocampus in *Kcnk9*KO^{mat} hybrid mice treated with CI-994 (n= 4) compared to *Kcnk9*KO^{mat} DMSO controls (n= 8). No difference between the maternal *Kcnk9* allele expression comparing DMSO (n= 9) and CI-994 (n=4) treated mice **(c)** Gene expression of *Kcnk9* in WT mice treated with DMSO compared to CI-994 treated mice shows no significant differences in several analysed brain regions. WT cerebellum (DMSO n= 9, CI-994 n=12), hypothalamus (DMSO n= 9, CI-994 n= 11) and hippocampus (DMSO n=14, CI-994 n=4). Mann-Whitney U, Values are means ± SEM. Statistical analyses and approaches are provided in Supplementary Table 1. Source data are provided as a Source Data file. Components of this figure were created using Servier Medical Art templates, which are licensed under a Creative Commons Attribution 3.0 Unported License; https://smart.servier.com.

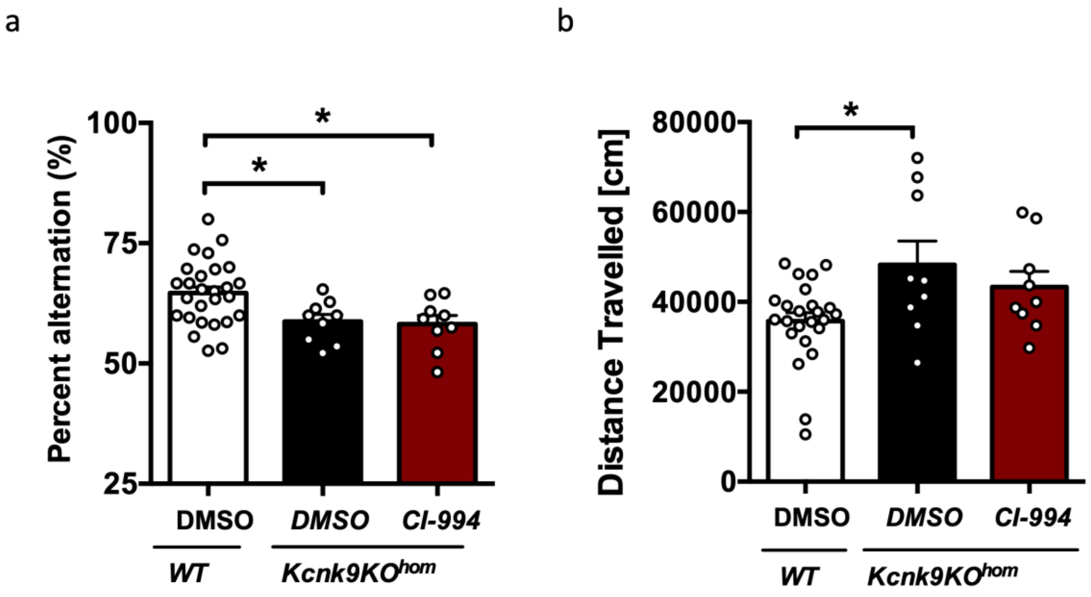

Supplementary Figure 5: No behavioural rescue of *Kcnk9*KO^{hom} mice after CI-994 treatment. (a) Y-maze percentage alternation analysis of DMSO-treated WT (n=27), DMSO-treated *Kcnk9*KO^{hom} (n=9) and CI994-treated *Kcnk9*KO^{hom} (n=9) treated mice. DMSO- and CI-994-treated *Kcnk9*KO^{hom} mice display a significant decrease in percentage alteration compared to WT mice. One-way ANOVA: $F(2, 39) = 4.710$, $P = 0.0147$; followed by Bonferroni's multiple comparison post hoc test, $*P < 0.05$. **(b)** Total locomotor activity in dark (12h) phase reveals no significant difference between DMSO-treated *Kcnk9*KO^{hom} (n=9) and CI-994-treated *Kcnk9*KO^{hom} (n=9) treated mice. A significant difference was observed by comparing DMSO-treated WT (n=24) and *Kcnk9*KO^{hom} mice. One-way ANOVA: $F(2, 42) = 5.569$, $P = 0.0072$; followed by Bonferroni's multiple comparison post hoc test, $*P < 0.05$. **(a-b)** Behavioral data are from biologically independent animals (n= number of mice). Values are means ± SEM Statistical analyses and approaches are provided in Supplementary Table 1. Source data are provided as a Source Data file.

.

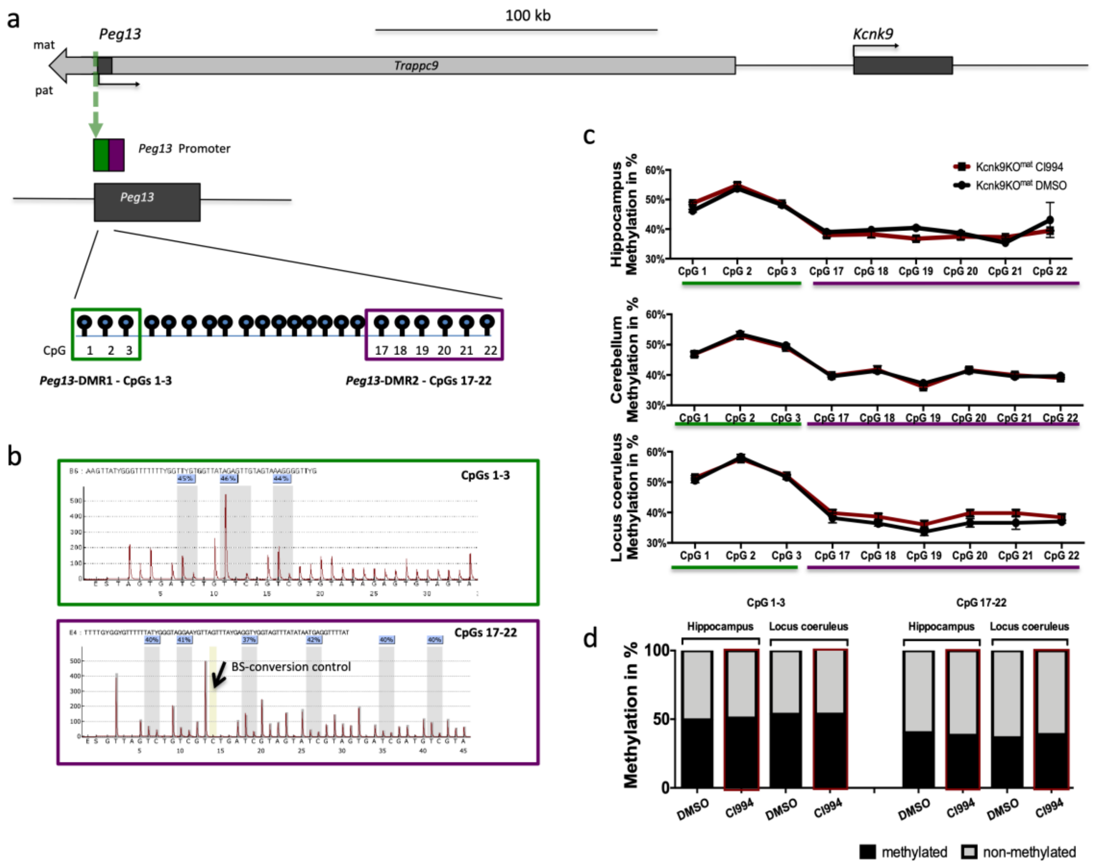

Supplementary Figure 6: CI-994 treatment of *Kcnk9*KO^{mat} mice does not interfere with DNA methylation at the *Peg13*-DMR. (a) Schematic presentation of the *Kcnk9* and *Peg13* loci on distal mouse chromosome 15. The *Peg13* differentially methylated region (*Peg13*-DMR) is analyzed in two separate assays. *Peg13*-DMR1 (green) and *Peg13*-DMR2 (violet). Individual CpGs analysed are depicted as lollipops. **(b)** The pyrogram shows the methylation status of the *Peg13*-DMRs in the hippocampus. The Y-axis shows the intensity of the light emission. The X-axis shows the dispensing order of the added nucleotides. The CpGs are highlighted in gray. In the blue box, the percentage of methylation is given. The bisulfite conversion control is highlighted in yellow. **(c,d)** CI-994 did not significantly alter the DNA methylation status of *Peg13*-DMR1 (CpGs 1-3) and *Peg13*-DMR2 (CpGs 17-22) in hippocampus, cerebellum or locus coeruleus measured using the bisulfite pyrosequencing method, n=3-5 DNA samples/ group, biologically independent animals. Values are means ± SEM. Statistical analyses and approaches are provided in Supplementary Table 1. Source data are provided as a Source Data file.

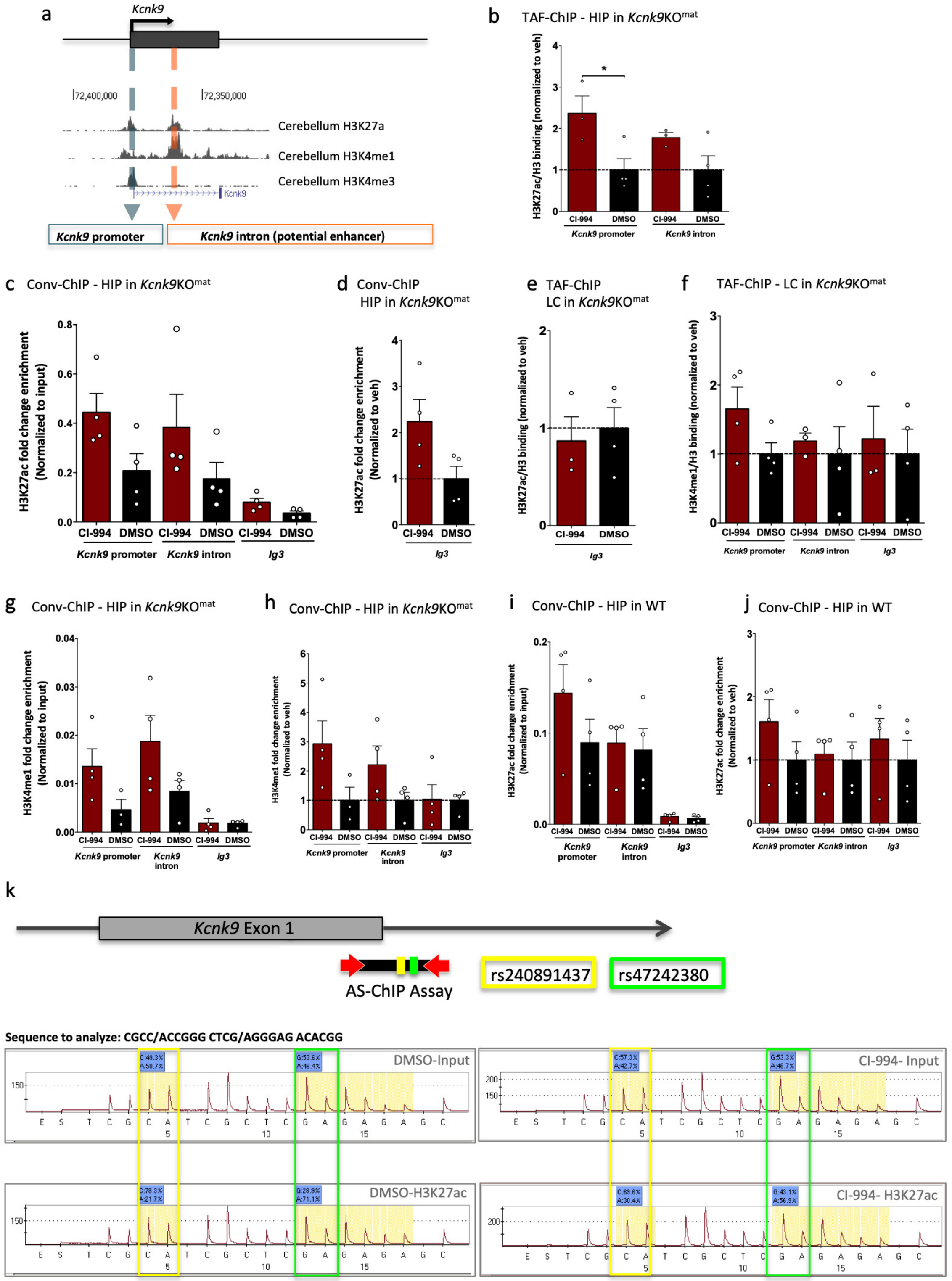

Supplementary Figure 7: CI-994 treatment of *Kcnk9*KO^{mat} mice affects histone modifications at the *Kcnk9* locus. (a) Schematic presentation of *Kcnk9* on distal mouse chromosome 15. The murine *Kcnk9* gene is shown with the corresponding H3K27ac, H3K4me1 and H3K4me3 peaks (UCSC Genome Browser on Mouse July 2007 (NCBI37/mm9) Assembly). **(b)** TAF-ChIP-qPCR of H3K27ac marks at the promoter and intronic region of *Kcnk9* in the hippocampus of *Kcnk9*KO^{mat}animals following treatment with CI-994 (normalized to veh), *Kcnk9* promoter: P = 0.0342, and *Kcnk9* intron $P = 0.1172$, each DMSO vs. CI-994 **(c)** TAF-ChIP-qPCR of H3K27ac marks at the promoter and intronic region of *Kcnk9* in the hippocampus of *Kcnk9*KO^{mat} animals following treatment with CI-994 (% of input). Related to figure 6b. **(d)** H3K27ac enrichment at intergenic control region in the hippocampus of *Kcnk9*KO^{mat} animals following treatment with CI-994 (normalized to veh). Related to figure 6b and suppl. figure 8c. **(e)** H3K27ac enrichment (norm to H3) at intergenic control region in the locus coeruleus of *Kcnk9*KO^{mat} animals following treatment with CI-994 (normalized to veh). Related to figure 6b. **(f)** TAF-ChIP-qPCR of H3K4me1 marks at the promoter and intronic region of *Kcnk9* in locus coeruleus of *Kcnk9*KO^{mat}animals following

treatment with CI-994 (normalized to veh). **(g-h)** Conventional ChIP-qPCR of H3K4me1 marks at the promoter and intronic region of *Kcnk9* in the hippocampus of *Kcnk9*KO^{mat} animals following treatment with CI-994 presented as (g) % of input and (h) normalized to veh. **(i-j)** Conventional ChIP-qPCR of H3K27ac marks at the promoter and intronic region of *Kcnk9* in the hippocampus of wildtype C57BL/6 animals following treatment with CI-994 presented as (i) % of input and (j) normalized to veh. **(b-j)** Values are means ± SEM, Student's t-test; (n=3-4 animals/group). n= 3-4 samples/group, biologically independent animals. Data per plot was generated in 2 independent experiments. **(k)** Allele-specific ChIP-qPCR. The pyrogram shows the status of SNPS rs240891437 and rs47242380 in the intronic region of *Kcnk9* in the hippocampus. The Y-axis shows the intensity of the light emission. The X-axis shows the dispensing order of the added nucleotides. In the blue box, the percentage of the nucleotide arising from maternal and paternal allele is given. Statistical analyses and approaches are provided in Supplementary Table 1. Source data are provided as a Source Data file.

Supplementary Table 1: Summary of statistical analyses and approaches

Subject	Figure	Genotype	Mean ± SEM	Factor	n	Statistics	Interaction	F value	P value	post hoc	adjusted p-values
Y-maze alteration	1b	WT	64.31 ± 1.619		23	one way ANOVA		F (2, 91) = 7.261	P=0.0012	Bonferroni's multiple comparisons test	***P<0.01 (WT vs. Kcnk 9KO^{mat})* **P<0.05 (WT vs. Kcnk 9KO^{hom})*
		Kcnk 9KO^{mat}	56.62 ± 1.438		27						
		Kcnk 9KO^{hom}	59.02 ± 1.020		44						
Circadian Rhythm	1c	WT	22685 ± 2059	Light phase	13	one way ANOVA		F (2, 29) = 2.281	P = 0.1203	Bonferroni's multiple comparisons test	n.s.
		Kcnk 9KO^{mat}	29678 ± 3623		10						
		Kcnk 9KO^{hom}	30978 ± 3817		9						
		WT	38741 ± 2384	Dark phase	18	one way ANOVA		F (2, 43) = 22.70	P < 0.0001	Bonferroni's multiple comparisons test	**P<0.05 (WT vs. Kcnk 9KO^{mat})* *****P<0.0001 (WT vs. Kcnk 9KO^{hom})* ***P<0.01 (Kcnk9KO^{mat} vs. Kcnk 9KO^{hom})*
		Kcnk 9KO^{mat}	48170 ± 2457		13						
		Kcnk 9KO^{hom}	61800 ± 2627		15						
QUASEP	1d	Maternal expression of (C57BL/6xCast/Ei)F1	96,12142 ± 0,171	Cerebellum	14						
			99,15 ± 0,298	Pons	10						
			96,225 ± 1,541	Olfactory bulb	12						
			93,22143 ± 0,334	Cortex	14						
			99,45715 ± 0,204	Hippocampus	14						
			98,43333 ± 0,358	Hypothalamus	9						
			95,28 ± 0,360	Striatum	5						
			99,06 ± 0,434	Midbrain	5						
			98,38 ± 0,450	Medulla	5						
			86,5 ± 1,843	Locus coeruleus	4						
		Paternal expression of (C57BL/6xCast/Ei)F1	3,939 ± 0,154	Cerebellum	14						
			0,850 ± 0,298	Pons	10						
			3,775 ± 1,541	Olfactory bulb	12						
			6,750 ± 0,314	Cortex	14						
			0,543 ± 0,204	Hippocampus	14						
			1,567 ± 0,358	Hypothalamus	9						
			4,720 ± 0,360	Striatum	5						
			0,940 ± 0,434	Midbrain	5						
			1,620 ± 0,450	Medulla	5						
			13,500 ± 1,843	Locus coeruleus	4						
Open field (percent in center)	Suppl. 1a	WT	14.00 ± 1.704		12	one way ANOVA		F (2, 44) = 0.3167	P = 0.7302	Bonferroni's multiple comparisons test	n.s.
		Kcnk 9KO^{mat}	17.97 ± 1.895		16						
		Kcnk 9KO^{hom}	13.11 ± 1.361		19						
Elevated plus maze (duration in open arms)	Suppl. 1b	WT	14.00 ± 1.704		22	one way ANOVA		F (2, 81) = 2.568	P = 0.0829	Bonferroni's multiple comparisons test	n.s.
		Kcnk 9KO^{mat}	17.97 ± 1.895		27						
		Kcnk 9KO^{hom}	13.11 ± 1.361		35						
Rotarod (Latency to fall)	Suppl. 1c	WT	240.5 ± 14.59		15	one way ANOVA		F (2, 55) = 0.01168	P = 0.9884	Bonferroni's multiple comparisons test	n.s.
		Kcnk 9KO^{mat}	237.6 ± 15.72		12						
		Kcnk 9KO^{hom}	240.4 ± 10.49		31						
relative *Kcnk9* expression after knock-down	Fig. 2b	shRNA 1	0,250		1	**Arithmetic means of *Kcnk9* expression of presented IDs were provided by Sirion Biotech**					
		shRNA 2	0,080		1						
		shRNA 3	0,110		1						
		shRNA 4	0,070		1						
		shRNA 5	0,160		1						
		shRNA 6	0,030		1						
		shRNA 7	0,060		1						
		shRNA 8	0,040		1						
		shRNA 9	0,050		1						
		shRNA 10	0,060		1						
		neagtive control	1,000		1						
Kcnk9 expression after knock-down	Fig. 2c left	WT scrambled control	1.000 ± 0.05220	PFC	8	Mann-Whitney U (Two-tailed)			n.s.		
		WT *kcnk9* knock-down	1.031 ± 0.04726		4						
		WT scrambled control	1.000 ± 0.08301	Hippoccampus	7				n.s.		
		WT *kcnk9* knock-down	0.9579 ± 0.06216		4						
		WT scrambled control	1.000 ± 0.1570	Locus coeruleus	7				P = 0,004		
		WT *kcnk9* knock-down	0.2067 ± 0.08371		4						
Tyrosine hydroxylase expression	Fig. 2c right	WT scrambled control	1.148 ± 0.1343	Locus coeruleus	7	Mann-Whitney U (Two-tailed)			n.s.		
		WT *kcnk9* knock-down	1.000 ± 0.1703		4						

**Circadian Rhythm**	**Fig. 2d**	WT scrambled control	78517 ± 5343	Dark phase	9	**Mann-Whitney U (Two-tailed)**		P=0,0101		
		WT *kcnk9* knock-down	105984 ± 6699		10					
**Y-maze alteration**	**Fig. 2e**	WT scrambled control	60.44 ± 2.352		9	**Mann-Whitney U (Two-tailed)**		P=0,0797		
		WT *kcnk9* knock-down	53.20 ± 4.002		10					
**Tyrosine hydroxylase expression**	**Suppl. 2a**	*Kcnk* 9KO mat	420,600 ± 24,150	LC	6	**one way ANOVA**	F (2, 13) = 242.0			Locus Coeruleus vs. Cerebellum ****P < 0.0001
			6,562 ± 1,117	Cerebellum	5					Locus Coeruleus vs. Hippocampus ****P < 0.0001
			0,664 ± 0.1037	Hippocampus	5					Cerebellum vs. Hippocampus
***Kcnk9* expression**	**Suppl. 2c**	Kcnk9KOmat	1,000 ± 0,637	LC	3	**Mann-Whitney U (Two-tailed)**		P=0.1000		
		WT	5,914 ± 0,033		3					
**Mean frequency**	**3d**	WT	2,383 ± 0,153	day	46	**2-way-ANOVA**	Interaction F (2, 182) = 4.435	P = 0.0132	Bonferroni's multiple comparisons test	Light Phase:Kcnk9KOhom vs. Dark phase:Kcnk9KOhom **P<0.01
		Kcnk 9KO mat	2,593 ± 0,181	day	41					
		Kcnk 9KO hom	2,350 ± 0,258	day	22		Phase F (1, 182) = 16.93	P < 0.0001		
		WT	3,265 ± 0,256	night	23					Dark phase:Kcnk9KOmat vs. Dark phase:Kcnk9KOhom *P<0.05
		Kcnk 9KO mat	2,640 ± 0,284	night	26					
		Kcnk 9KO hom	3,799 ± 0,295	night	30		Genotype F (2, 182) = 1.836	P = 0.1624		
**Cortical neurons all drugs**	**4c**				Cultures					vs. DMSO
		Kcnk 9KO mat	1.000 ± 0.0417	DMSO	7	**one way ANOVA**	F (4, 14) = 92.00	P < 0.0001	Bonferroni's multiple comparisons test	
			3.853 ± 0.3724	VPA	3					****P < 0.0001
			5.307 ± 0.6183	DZnep	3					****P < 0.0001
			5.963 ± 0.1874	SAHA	3					****P < 0.0001
			6.738 ± 0.2760	CI-994	3					****P < 0.0001
**Cortical neurons CI-994**	**4d**				Cultures					*Kcnk* 9KO mat
		Kcnk 9KO mat	1.000 ± 0.0656	DMSO	9	**one way ANOVA**	F (5, 22) = 75.51	P < 0.0001	Bonferroni's multiple comparisons test	vs. DMSO
			2.586 ± 0.3351	4 µM	2					***P < 0.001
			3.642 ± 0.2920	20 µM	4					****P < 0.0001
			4.349 ± 0.1035	40 µM	4					****P < 0.0001
			5.216 ± 0.2264	80 µM	4					****P < 0.0001
			3.636 ± 0.2824	WT DMSO	5					****P < 0.0001
**Duration of unsilencing in cortical neurons**	**4e**			(3-5 wells/sample)						
		Kcnk 9KO mat	1,000 ± 0,104	day 1 DMSO	2 samples	**Unpaired t-test (Two-tailed)**		1 day P=0,0297		
			2,738 ± 0,339	day 1 CI-994	3 samples					
			1,000 ± 0,089	day 10 DMSO	6 samples			10 days P=0.0001		
			2,619 ± 0,265	day 10 CI-994	4 samples					
**Cortical neurons drugs no effect**	**Suppl. 3a**				Cultures			vs. DMSO		
		Kcnk 9KO mat	1.000 ± 0.0417	DMSO	7	**Mann-Whitney U (Two-tailed)**				
			0.901 ± 0.013	Zebularine	3			P= 0.0667		
			0.548 ± 0.338	C646	4			P =0.4727		
**RT-qPCR analysis of cluster genes**	**Suppl. 3b**	*Kcnk* 9KO mat			Cultures	**Mann-Whitney U**				
		Eif2c2	1,050 ± 0,014	CI-994 20 µM	2					
		Chrac1	0,646 ± 0,014		3					
		Peg13	0,442 ± 0,018		3					
		Trappc9	0,337 ± 0,678		3					
		Kcnk9	6,448 ± 0,016		2					
		Eif2c2	2,718 ± 0,024	CI-994 80 µM	3					
		Chrac1	1,084 ± 0,024		2					
		Peg13	1,060 ± 0,024		3					
		Trappc9	0,965 ± 0,608		3					
		Kcnk9	20,870 ± 0,042		2					
**Neuron density after CI-994 treatment**	**Suppl. 3d**	*Kcnk* 9KO mat	41,000 ± 4,082	DMSO	12	**one way ANOVA**	F (2, 27) = 0.8983	P = 0.4191		
			34,580 ± 3,530	CI-994 10 µM	9					
			34,860 ± 4,035	CI-994 20 µM	9					
**RT-qPCR *Kcnk9* expression in several brain regions**	**5b**	*Kcnk* 9KO mat	2.89 ± 0,20	Cerebellum	DMSO: 12 CI-994: 13	**Mann-Whitney U (Two-tailed)**		P < 0.0001		
			1,18 ± 0,21	Cortex	DMSO: 6 CI-994: 9			P= 0,3251		
			2.65 ± 0.55	Hippocampus	DMSO: 12 CI-994: 14			P= 0.0003		
			2,22 ± 0,55	Pons	DMSO: 7 CI-994: 8			P= 0,0002		
			2,77 ± 0,41	Hypothalamus	DMSO: 9 CI-994: 12			P= 0,0003		
			2,01 ± 1,07	Medulla	DMSO: 6 CI-994: 6			P= 0,3961		
			0,96 ± 0,19	Prefrontal cortex	DMSO: 9 CI-994: 9			P= 0,7319		
			2.08 ± 0.28	Olfactory bulb	DMSO: 6			P= 0.0022		

			[illegible]	[illegible]					[illegible]		
					CI-994: 6						
			2,90 ± 0,92	Locus coeruleus	DMSO: 7						
					CI-994: 8				P= 0,0401		
			2,25 ± 0,69	Caudal Pontine Reticular Formation	DMSO: 7						
					CI-994: 4				P= 0,1409		
**Cerebellum after CI-994 treatment**	**5c**	WT	5.337 ± 0.165	DMSO	9	**2-way-ANOVA**	Interaction	F (1, 42) = 26.16	P < 0.0001	Bonferroni's multiple comparisons test	****P<0.0001 (all comparisons except DMSO: WT vs CI-994: WT)
			5.445 ± 0.123	CI-994	12		Treatment	F (1, 42) = 34.92	P < 0.0001		
		Kcnk 9KO^{mat}	1.000 ± 0.125	DMSO	12						
			2.497 ± 0.133	CI-994	13		Genotype	F (1, 42) = 719.1	P < 0.0001		
**RT-qPCR analysis of cluster genes**	**5d**	*Kcnk* 9KO^{mat}			Hippocampus	**Mann-Whitney U**			*Kcnk9* CI-994 vs. DMSO: P=0.0079		
		Eif2c2	1,000 ± 0,016	DMSO	5						
		Chrac1	1,000 ± 0,044	DMSO	5						
		Peg13	1,000 ± 0,032	DMSO	5						
		Trappc9	1,000 ± 0,054	DMSO	5						
		Kcnk9	1,000 ± 0,091	DMSO	5						
		Eif2c2	1,000 ± 0,156	CI994 (30mg/kg)	5						
		Chrac1	0,982 ± 0,088	CI994 (30mg/kg)	5						
		Peg13	0,986 ± 0,105	CI994 (30mg/kg)	5						
		Trappc9	1,210 ± 0,188	CI994 (30mg/kg)	5						
		Kcnk9	2,313 ± 0,423	CI994 (30mg/kg)	5						
**Ymaze alteration after CI-994 treatment**	**5e**	WT	64.62772 ± 1.290	DMSO	20	**2-way-ANOVA**	Interaction	F (1, 96) = 3.700	P = 0.0574	Tukey multiple comparisons test	*****P<0.01*** *(WT:DMSO vs. Kcnk* 9KO^{mat} :DMSO)
			64.77955 ±1.614	CI-994	18		Treatment	F (1, 96) = 4.096	P = 0.0458		**P<0.01 (WT:CI-994 vs. Kcnk9KOmat :DMSO)
		Kcnk 9KO^{mat}	57.179977 ± 1.584	DMSO	21			F (1, 96) = 8.992	P = 0.0035		
			63.15288 ± 1.493	CI-994	24		Genotype				*P<0.05 (Kcnk9KOmat: CI-994 vs. Kcnk9KOmat :DMSO)
**Circadian Rhythm light phase CI-994**	**5f**	WT	17562,000 ± 999,600	DMSO	20	**2-way-ANOVA**	Interaction	F (1, 43) = 0.5104	P = 0.0018	Bonferroni's multiple comparisons test	n.s.
			16754,000 ± 1061,000	CI-994	10		Treatment	F (1, 43) = 0.006126	P = 0.0366		
		Kcnk 9KO^{mat}	15950,000 ± 1073,000	DMSO	8						
			16958,000 ± 1557,000	CI-994	9		Genotype	F (1, 43) = 0.3064	P = 0.1763		
**Circadian Rhythm dark phase CI-994**	**5f**	WT	35719,000 ± 1874,000	DMSO	24	**2-way-ANOVA**	Interaction	F (1, 60) = 10.68	P = 0.0031	Bonferroni's multiple comparisons test	*****P<0.01*** *(WT:DMSO vs. Kcnk* 9KO^{mat} :DMSO)
			38601,000 ± 2630,000	CI-994	15		Treatment	F (1, 60) = 4.571	P = 0.0518		
		Kcnk 9KO^{mat}	47535,000 ± 3016,000	DMSO	12						**P<0.01 (*Kcnk9* KOmat: CI-994 vs. *Kcnk9* KOmat :DMSO)
			33758,000 ± 2785,000	CI-994	13		Genotype	F (1, 60) = 1.872	P = 0.1960		
**Allele-specific expression of *Kcnk9***	**Suppl. 4b**	WT hippocampus	1,000 ± 0,185	maternal DMSO	8	**Mann-Whitney U**			P=0,6828		
			1,296 ± 0,314	maternal CI-994	4						
			1,000 ± 0,268	paternal DMSO	9				P=0,2601		
			3,583 ± 1,626	paternal CI-994	4						
***Kcnk9* expression after CI-994 treatment**	**Suppl. 4c**	WT cerebellum	1,000 ± 0,128	DMSO	9	**Mann-Whitney U**			P=0,3368		
			1,054 ± 0,076	CI-994	12						
		WT hypothalamus	1,000 ± 0,054	DMSO	9				P=0,1726		
			1,127 ± 0,104	CI-994	11						
		WT hippocampus	1,000 ± 0,078	DMSO	14				P=0,8778		
			1,103 ± 0,274	CI-994	4						
**Y-maze alteration**	**Suppl.5a**	WT	64,630 ± 1,291	DMSO	27	**one way ANOVA**		F (2, 39) = 4.710	P = 0.0147	Bonferroni's multiple comparisons test	**P<0.05 (WT DMSO vs. Kcnk* 9KO^{ma}DMSO)
		Kcnk9KO^{hom}	58,740 ± 1,465	DMSO	9						
		Kcnk 9KO^{hom}	58,200 ± 1,775	CI-994	9						
**Circadian Rhythm**	**Suppl. 5b**	WT	35719,000 ± 1874,000	DMSO	24	**one way ANOVA**		F (2, 42) = 5.569	P = 0.0072	Bonferroni's multiple comparisons test	**P<0.05 (WT DMSO vs. Kcnk* 9KO^{ma}DMSO)
		Kcnk9KO^{hom}	48275,000 ± 5271,000	DMSO	9						**P<0.05 (WT DMSO vs. Kcnk* 9KO^{ma} CI-994
		Kcnk 9KO^{hom}	43338,000 ± 3433,000	CI-994	9						
**Conventional ChIP H3K27ac**	**6b**	H3K27ac fold enrichment in hippocampus (Normalized to Veh)	1,000 ± 0,147	*kcnk9* prom	DMSO: 9	**Student's t-test (Two-tailed)**			P = 0.0001		
			2,137 ± 0,1687	*kcnk9* prom	CI-994: 9						
			1,000 ± 0,232	*kcnk9* intron	DMSO: 8				P = 0.0270		
			2,097 ± 0,3791	*kcnk9* intron	CI-994: 8						
**Low-input ChIP H3K27ac**		H3K27ac/H3 binding in LC (Fold Change to Veh)	1.000 ± 0.1245	*kcnk9* prom	DMSO: 4				P = 0.0450		
			2.288 ± 0.4945	*kcnk9* prom	CI-994: 4						
			1.000 ± 0.2305	*kcnk9* intron	DMSO: 4				P =0.0202		
			2.414 ± 0.3838	*kcnk9* intron	CI-994: 3						
			72,990		WT DMSO=3				P =0,2		
			63,942		WT CI-994=4						

Allele-specific ChIP	6c	Maternal H3K27ac enrichment in the hippocampus (C57BL/6xCast/Ei)F1	68,505			rs 240891437	*Kcnk* 9KO^{mat} :DMSO=6	Student's t-test (Two-tailed)	P =0.4476
			61,894				*Kcnk* 9KO^{mat} :CI-994=4		
			61,180			rs 47242380	WT DMSO=3		P =0.0571
			51,170				WT CI-994=4		
			57,820				*Kcnk* 9KO^{mat} :DMSO=6		P =0.0476
			51,360				*Kcnk* 9KO^{mat} :CI-994=4		
		Paternal H3K27ac enrichment in the hippocampus (C57BL/6xCast/Ei)F1	27,010			rs 240891437	WT DMSO=3	Student's t-test (Two-tailed)	P =0,2
			36,058				WT CI-994=4		
			31,495				*Kcnk* 9KO^{mat} :DMSO=6		P =0.4476
			38,106				*Kcnk* 9KO^{mat} :CI-994=4		
			38,800			rs 47242380	WT DMSO=3		P =0.0571
			48,830				WT CI-994=4		
			42,183				*Kcnk* 9KO^{mat} :DMSO=6		P =0.0476
			48,640				*Kcnk* 9KO^{mat} :CI-994=4		
Methylation of Peg13-DMR	Suppl. 6	Hippocampus	49,37	±	0,65	CpGs 1-3	DMSO: 3	RM 2-way-ANOVA treated vs non-treated	P= 0.1348
			50,71	±	0,59		CI-994: 3		
			39,36	±	1,34	CpGs 17-22	DMSO: 3		P= 0.2744
			37,87	±	2,67		CI-994: 3		
		Cerebellum	50,05	±	0,71	CpGs 1-3	DMSO: 4		P= 0.6190
			49,61	±	0,51		CI-994: 3		
			39,77	±	0,36	CpGs 17-22	DMSO: 4		P= 0.9366
			39,72	±	0,70		CI-994: 3		
		LC	53,47	±	0,81	CpGs 1-3	DMSO: 5		P= 0.7135
			53,72	±	0,62		CI-994: 5		
			36,40	±	1,30	CpGs 17-22	DMSO: 5		P= 0.1840
			38,73	±	1,18		CI-994: 5		
TAF-ChIP H3K27ac	Suppl. 7b	*H3K27ac/H3 binding (normalized to veh) in hippocampus of Kcnk* 9KO^{mat}	1.000 ± 0.2726			*kcnk9* prom	DMSO: 4	Student's t-test	P = 0.0342
			2.369 ± 0.4146				CI-994: 3		
			1.000 ± 0.3419			*kcnk9* intron	DMSO: 4		P = 0.1172
			1.787 ± 0.1179				CI-994: 3		
Convent. ChIP H3K27ac	Suppl. 7c	H3K27ac fold change enrichment (Normalized to input) *in hippocampus of Kcnk* 9KO^{mat}	0,4436	±	0,07741	*kcnk9* prom	CI-994: 4	Student's t-test (Two-tailed)	P =0,0648
			0,2074	±	0,07044		DMSO: 4		
			0,3834	±	0,1338	*kcnk9* intron	CI-994: 4		P = 0.2130
			0,176	±	0,0652		DMSO: 4		
			0,0796	±	0,01726	*Ig3*	CI-994: 4		P =0.0671
			0,03558	±	0,009545		DMSO: 4		
Convent. ChIP H3K27ac	Suppl. 7d	H3K27ac fold change enrichment (normalized to veh) in hippocampus	2,237	±	0,485	*Ig3*	CI-994: 4	Student's t-test (Two-tailed)	P=0,0671
			1,000	±	0,268		DMSO: 4		
TAF-ChIP H3K27ac	Suppl. 7e	H3K27ac/H3 binding (normalized to veh) in LC	0,869		0,248	*Ig3*	CI-994: 3	Student's t-test (Two-tailed)	P=0,7046
			1,000		0,212		DMSO: 4		
Low-input ChIP H3K4me1	Suppl. 7f	H3K4me1/H3 binding (normalized to veh) in LC of *Kcnk* 9KO^{mat}	1,656	±	0,313	*kcnk9* prom	CI-994: 4	Student's t-test (Two-tailed)	P = 0.1118
			1,000	±	0,162		DMSO: 4		
			1,188	±	0,116	*kcnk9* intron	CI-994: 3		P = 0.7092
			1	±	0,3949		DMSO: 4		
			1,219	±	0,473	*Ig3*	CI-994: 3		P =0.7226
			1	±	0,3612		DMSO: 4		
Convent. ChIP H3K4me1	Suppl. 7g	H3K4me1 fold change enrichment (Normalized to input) *in Kcnk* 9KO^{mat} hippocampus	0,014	±	0,004	*kcnk9* prom	CI-994: 4	Student's t-test (Two-tailed)	P = 0.1115
			0,005	±	0,002		DMSO: 3		
			0,019	±	0,005	*kcnk9* intron	CI-994: 4		P =0,1299
			0,00846	±	0,002263		DMSO: 4		
			0,002	±	0,001	*Ig3*	CI-994: 4		P =0.9485
			0,001843	±	0,0003437		DMSO: 4		
Convent. ChIP H3K4me1	Suppl. 7h	H3K4me1 fold change enrichment (Normalized to veh) in hippocampus of *Kcnk* 9KO^{mat}	2,930	±	0,782	*kcnk9* prom	CI-994: 4	Student's t-test (Two-tailed)	P = 0.1115
			1,000	±	0,453		DMSO: 3		
			2,217	±	0,640	*kcnk9* intron	CI-994: 4		P =0,1299
			1	±	0,2676		DMSO: 4		
			1,036	±	0,504	*Ig3*	CI-994: 4		P =0.9485
			1	±	0,1865		DMSO: 4		
Convent. ChIP H3K27ac	Suppl. 7i	H3K27ac fold change enrichment (Normalized to input) in hippocampus of WT	0,144	±	0,031	*kcnk9* prom	CI-994: 4	Student's t-test (Two-tailed)	P = 0.2312
			0,089	±	0,026		DMSO: 4		
			0,089	±	0,017	*kcnk9* intron	CI-994: 4		P =0.7998
			0,0816	±	0,02327		DMSO: 4		
			0,009	±	0,002	*Ig3*	CI-994: 4		P =0.4922

			0,006498	±	0,002042		DMSO: 4		
**Convent. ChIP H3K27ac**	**Suppl. 7j**	H3K27ac fold change enrichment (Normalized to veh) in hippocampus of WT	1,606	±	0,351	*kcnk9* prom	CI-994: 4	**Student's t-test (Two-tailed)**	P =0,2312
			1,000	±	0,290		DMSO: 4		
			1,094	±	0,210	*kcnk9* intron	CI-994: 4		P =0.7998
			1	±	0,2851		DMSO: 4		
			1,331	±	0,325	Ig3	CI-994: 4		P = 0.7092
			1	±	0,3143		DMSO: 4		

Abbreviations: n, number of samples/mice; n.s., not significant

Supplementary Table 2: Primers used for PCR and pyrosequencing analyses

**Primer**			**Sequence (5‘ – 3‘)**
TASK3-P3	F	Genotyping	TGCGAGCTTCAGAGAGGATG
TASK3-P4	R	Genotyping	ATGCTCTAATCTCCAGTCTG
Kcnk9 Exon2	F	Genotyping	CACCACGCCATGTACTTCCT
Kcnk9 Exon2	R	Genotyping	GGACCGGAAGTAGGTGTTCC
Kcnk9-SNP	F	Allele-specific RT-qPCR	CACAACTATCGGATATGGACATGC
Kcnk9-SNP	R	Allele-specific RT-qPCR	TGCCGCGGTGTTCGAT
Kcnk477	F	QUASEP	GCCTGTACCTTCACCTAC
Kcnk477	R	QUASEP	CACAACTATCGGATATGGACATGC
Kcnk477_S	Seq	QUASEP	TGCCGCGGTGTTC
kcnk9-283/284	F	RT-qPCR	ACTATCGGATATGGACATGCTGC
kcnk9-283/284	R	RT-qPCR	GCCCAGGCTCTGGAACATAA
Bdnf	F	RT-qPCR	ATCCACTGAGCAAAGCCGAA
Bdnf	R	RT-qPCR	CCTGGTGGAACATTGTGGCT
Impa2	F	RT-qPCR	CGTGCGGGACAAATCATCAG
Impa2	R	RT-qPCR	AAGGAAACCGCTTTCGCAAC
Kcnk9 promoter	F	ChIP-qPCR	CGTGTGCGCTACATCTCCTA
Kcnk9 promoter	R	ChIP-qPCR	ATTCGCCGGTTCCTTCTACT
Kcnk9 intron	F	ChIP-qPCR	AGGGCAGATGCTTAAGAGGA
Kcnk9 intron	R	ChIP-qPCR	CATCTGTTCTGTACCCCATCC
mPeg13-CpG 1-9	F	Methylation analysis	TTGGATGAGTTATTATATAAGGTTTAAAA
mPeg13-CpG 1-9	R	Methylation analysis	ACAACTACCTACATTCCAAATCT
mPeg13-CpG_1-9	Seq	Methylation analysis	AAATTTTAATAAGATGGGTTAAT
mPeg13-CpG 17-22	F	Methylation analysis	AGATTTGGAATGTAGGTAGTTGTGA
mPeg13-CpG 17-22	R	Methylation analysis	CCTCAATAAAACCATTCTAATCAACTAT
mPeg13-CpG 17-22	Seq	Methylation analysis	GGTAATTTGTTAGGTGGAGATATA
Ago2_F	F	Cluster gene analysis	CGACAACATCACCCATCCCA
Ago2_R	R	Cluster gene analysis	TTTGATTGTTCTCCCGGTGGT
Chrac1_F	F	Cluster gene analysis	AAGAGCTCTCCCGAGGTGTC
Chrac1-R	R	Cluster gene analysis	TACTGAACAAAGAGCTCCGTGGC
Peg13_F	F	Cluster gene analysis	AAGATCCGCGGCCTTTACTC
Peg13_R	R	Cluster gene analysis	TTTTGCCCATTCCTCGGTCA
Trappc9_F	F	Cluster gene analysis	TGGGGCTGAAAAGACACTACAA
Trappc9_R	R	Cluster gene analysis	TGGTAGTGTACCAGGGCGT
Kcnk9-SNP_EP-C_F	F	AS-RT-qPCR	TGCCGCGGTGTTCGAC
Kcnk9-SNP_EP_R	R	AS-RT-qPCR	GCATGTCCATATCCGATAGTTGTG
Kcnk9-SNP_EP-T_F	F	AS-RT-qPCR	TGCCGCGGTGTTCGAT
KCNK9-AS-ChIP-F	F	AS-CHIP	AAATTCGCCGGTTCCTTCTAC
KCNK9-AS-ChIP-R	R	AS-CHIP	gagatgtagcgcacacgaagc
KCNK9-AS-ChIP-S	Seq	AS-CHIP	aaggaatgggtgtgc

Abbreviations: F, forward primer; R, reverse primer; Seq, pyrosequencing primer

Supplementary References

1. Paxinos, G. & Franklin, K. B. J. The Mouse Brain in Stereotaxic Coordinates (Academic Press, 2008).
