# Supplementary material for: Inhibition of histone deacetylation rescues phenotype in a mouse model of Birk-Barel intellectual disability syndrome
Source: Nat Commun. 2020 Jan 24;11:480. doi: 10.1038/s41467-019-13918-4 (PMC6981138; doi:10.1038/s41467-019-13918-4)
Supplement: Supplementary file 2 — Reporting Summary [file 41467_2019_13918_MOESM2_ESM.pdf]

nature research

Corresponding author(s): DBPR NCOMMS-18-38021B

Last updated by author(s): Dec 1, 2019

Reporting Summary

Nature Research wishes to improve the reproducibility of the work that we publish. This form provides structure for consistency and transparency in reporting. For further information on Nature Research policies, see Authors & Referees and the Editorial Policy Checklist.

Statistics

For all statistical analyses, confirm that the following items are present in the figure legend, table legend, main text, or Methods section.

n/a	Confirmed	
☐	☒	The exact sample size (*n*) for each experimental group/condition, given as a discrete number and unit of measurement
☐	☒	A statement on whether measurements were taken from distinct samples or whether the same sample was measured repeatedly
☐	☒	The statistical test(s) used AND whether they are one- or two-sided<br>*Only common tests should be described solely by name; describe more complex techniques in the Methods section.*
☒	☐	A description of all covariates tested
☐	☒	A description of any assumptions or corrections, such as tests of normality and adjustment for multiple comparisons
☐	☒	A full description of the statistical parameters including central tendency (e.g. means) or other basic estimates (e.g. regression coefficient) AND variation (e.g. standard deviation) or associated estimates of uncertainty (e.g. confidence intervals)
☐	☒	For null hypothesis testing, the test statistic (e.g. F, t, r) with confidence intervals, effect sizes, degrees of freedom and P value noted<br>*Give P values as exact values whenever suitable.*
☒	☐	For Bayesian analysis, information on the choice of priors and Markov chain Monte Carlo settings
☒	☐	For hierarchical and complex designs, identification of the appropriate level for tests and full reporting of outcomes
☒	☐	Estimates of effect sizes (e.g. Cohen's *d*, Pearson's *r*), indicating how they were calculated

Our web collection on statistics for biologists contains articles on many of the points above.

Software and code

Policy information about availability of computer code

Data collection	StepOne Software v2.3; EthoVision XT 8; PyroMark ID 1.0 (database version 23.2); PyroMark CpG Software 1.0.11; HEKA Patchmaster 2x90.4; NIKON Imaging Software NIS-Elements 4.5
Data analysis	Graphpad Prism v7; StepOne Software v2.3; EthoVision XT 8; PyroMark ID 1.0 (database version 23.2); PyroMark CpG Software 1.0.11; HEKA Fitmaster 2x90.4; Wavemetrics IGOR Pro 8

For manuscripts utilizing custom algorithms or software that are central to the research but not yet described in published literature, software must be made available to editors/reviewers. We strongly encourage code deposition in a community repository (e.g. GitHub). See the Nature Research guidelines for submitting code & software for further information.

Data

Policy information about availability of data

All manuscripts must include a data availability statement. This statement should provide the following information, where applicable:

- Accession codes, unique identifiers, or web links for publicly available datasets
- A list of figures that have associated raw data
- A description of any restrictions on data availability

No restrictions on data availability

Field-specific reporting

Please select the one below that is the best fit for your research. If you are not sure, read the appropriate sections before making your selection.

☒ Life sciences ☐ Behavioural & social sciences ☐ Ecological, evolutionary & environmental sciences

Life sciences study design

All studies must disclose on these points even when the disclosure is negative.

Sample size

For behavioral experiments, a justifiable number of mice was used. Sample size evaluation based upon: Dell R.B., Holleran S., Ramakrishnan R. (2002), ILAR J. 2002;43(4):207-13.
For in vitro experiments, no statistical methods or calculations were used to predetermine sample size. The sample size was determined to be sufficient based on the size and consistency of the measurable differences between the groups.

Data exclusions

In general, outliers were identified with the Grubbs' test (Graphpad Prism). In the circadian rhythm experiments, several mice were excluded due to insufficient tracking in the light phase (low contrast between mouse and background). Tracking in the dark phase was overall successful (high contrast). One locus coeruleus sample due to insufficient tyrosine hydroxylase expression was excluded from analysis (Fig. 2c)

Replication

Mice from different litters and biological replicates were used in this study, unless stated otherwise.

Behavioral analysis: Experiments without injections of mice were performed in 7 individual sessions (Fig.1), experiments with injections of mice in 9 individual sessions (Fig.5)

RT-qPCR analysis: 3 technical replicates of one single sample were arithmetically averaged and integrated as one biological replicate in statistical analysis.

Randomization

Mice were initially genotyped and randomly divided into experimental groups with equal amount of mice per group. Male mice used in behavioral experiments were age-matched. Several breeding-dependent litters were utilized.

Blinding

Y-maze experiments and analyses were performed blinded in terms of genotype and treatment.

Circadian rhythm experiments were not performed blinded, but with an equal distribution of mice of different groups per run, which were analyzed simultaneously (restricted number of experimental setups). Several sessions were performed with all groups represented within each session.

Electrophysiological experiments were performed blinded.

In vitro experiments were not performed blinded. If investigators were not blinded to genotypes and treatment, the reported data were not subjective but rather computer-produced quantitative measurements.

Behavior experiments after AAV injection were performed blinded.

Reporting for specific materials, systems and methods

We require information from authors about some types of materials, experimental systems and methods used in many studies. Here, indicate whether each material, system or method listed is relevant to your study. If you are not sure if a list item applies to your research, read the appropriate section before selecting a response.

Materials & experimental systems

n/a	Involved in the study
☐	☒ Antibodies
☒	☐ Eukaryotic cell lines
☒	☐ Palaeontology
☐	☒ Animals and other organisms
☒	☐ Human research participants
☒	☐ Clinical data

Methods

n/a	Involved in the study
☒	☐ ChIP-seq
☒	☐ Flow cytometry
☒	☐ MRI-based neuroimaging

Antibodies

Antibodies used

All antibodies are commercially available:
Antigen/ Supplier/ Cat #
1. H3K27ac/ Abcam/ ab4729
2. H3K4me1/ Active motif/ 39297
3. Histone H3/ Abcam/ ab1791
4. Rabbit IgG/ Diagenode/ C15410206
5. Neuron-specific nuclear protein NeuN/ Abcam/ ab104225
6. AlexaFluor 488 anti-mouse/ Abcam / ab150077

	7. tyrosine hydroxylase/ Millipore/ MAB318<br>8. GFP/Life Technology/ A11122<br>9. Alexa Fluor 488 anti-rabbit/ Thermo Fisher Scientific, Invitrogen/ A11008<br>10. Alexa Fluor 568 anti-mouse/ Thermo Fisher Scientific, Invitrogen/ A11004
Validation	Validation for the antibodies are presented in relevant citations below or can be found on the manufacturer's website.<br>1. Nat Commun. 2018 Jan 11;9(1):161. doi: 10.1038/s41467-017-02536-7.<br>2. Cell. 2017 Oct 19;171(3):557-572.e24. doi: 10.1016/j.cell.2017.09.043.<br>3. Nat Commun. 2018 Jan 4;9(1):53. doi: 10.1038/s41467-017-02242-4.<br>4. Elife. 2018 Oct 4;7. pii: e33337. doi: 10.7554/eLife.33337.<br>5. J Neurosci. 2014 Oct 8;34(41):13586-99. doi: 10.1523/JNEUROSCI.5069-13.2014.<br>6. Sci Rep. 2017 Jan 25;7:41223. doi: 10.1038/srep41223.<br>7. J Biol Chem. 2003 Mar 14;278(11):9928-37. Epub 2003 Jan 2.

Animals and other organisms

Policy information about studies involving animals; ARRIVE guidelines recommended for reporting animal research

Laboratory animals	C57BL/6J and Mus musculus castaneus (Cast/Ei) mouse strains were used in this study. Details concerning genotype, sex, age and cross-breeding are described in the methods section or within individual experiments. For Kcnk9 knockout mouse generation, please see link : J Neurosci. 2009 Feb 25;29(8):2528-33. doi: 10.1523/JNEUROSCI.5764-08.2009. Heterozygous maternal Kcnk9 KO mice, homozygous Kcnk9 mice and WT were used in this study.
Wild animals	This study does not involve wild animals.
Field-collected samples	This study does not involve samples collected from the field.
Ethics oversight	All experimental procedures were performed in accordance with institutional animal welfare guidelines and were approved by the ethical committee of the state government of Rhineland-Palatinate, Germany.

Note that full information on the approval of the study protocol must also be provided in the manuscript.
